# Chromatin remodeling due to degradation of citrate carrier impairs osteogenesis of aged mesenchymal stem cells

**DOI:** 10.1038/s43587-021-00105-8

**Published:** 2021-09-13

**Authors:** Andromachi Pouikli, Swati Parekh, Monika Maleszewska, Chrysa Nikopoulou, Maarouf Baghdadi, Ignacio Tripodi, Kat Folz-Donahue, Yvonne Hinze, Andrea Mesaros, David Hoey, Patrick Giavalisco, Robin Dowell, Linda Partridge, Peter Tessarz

**Affiliations:** 1 Max-Planck Research Group Chromatin and Ageing, Max Planck Institute for Biology of Ageing, Cologne, Germany; 2 Department of Biological Mechanisms of Ageing, Max Planck Institute for Biology of Ageing, Cologne, Germany; 3 Computer Science, University of Colorado, Boulder, CO USA; 4 BioFrontiers Institute, University of Colorado, Boulder, CO USA; 5 FACS & Imaging Core Facility, Max Planck Institute for Biology of Ageing, Cologne, Germany; 6 Metabolomics Core Facility, Max Planck Institute for Biology of Ageing, Cologne, Germany; 7 Phenotyping Core Facility, Max Planck Institute for Biology of Ageing, Cologne, Germany; 8 Trinity Centre for Biomedical Engineering, Trinity Biomedical Sciences Institute, Trinity College Dublin, Dublin, Ireland; 9 Department of Mechanical, Manufacturing and Biomedical Engineering, School of Engineering, Trinity College Dublin, Dublin, Ireland; 10 Advanced Materials and Bioengineering Research Centre (AMBER), Royal College of Surgeons in Ireland and Trinity College Dublin, Dublin, Ireland; 11 Molecular, Cellular and Developmental Biology, University of Colorado, Boulder, CO USA; 12 Cologne Excellence Cluster on Stress Responses in Ageing-Associated Diseases (CECAD), Cologne, Germany

**Keywords:** Chromatin structure, Histone post-translational modifications, Ageing, Adult stem cells, Mitochondria

## Abstract

Aging is accompanied by a general decline in the function of many cellular pathways. However, whether these are causally or functionally interconnected remains elusive. Here, we study the effect of mitochondrial–nuclear communication on stem cell aging. We show that aged mesenchymal stem cells exhibit reduced chromatin accessibility and lower histone acetylation, particularly on promoters and enhancers of osteogenic genes. The reduced histone acetylation is due to impaired export of mitochondrial acetyl-CoA, owing to the lower levels of citrate carrier (CiC). We demonstrate that aged cells showed enhanced lysosomal degradation of CiC, which is mediated via mitochondrial-derived vesicles. Strikingly, restoring cytosolic acetyl-CoA levels either by exogenous CiC expression or via acetate supplementation, remodels the chromatin landscape and rescues the osteogenesis defects of aged mesenchymal stem cells. Collectively, our results establish a tight, age-dependent connection between mitochondrial quality control, chromatin and stem cell fate, which are linked together by CiC.

## Abstract (web summary)

Aged mesenchymal stem cells exhibit decreased osteogenesis. Pouikli et al. link impaired MSC differentiation to histone hypoacetylation caused by lower mitochondrial acetyl-CoA export due to enhanced lysosomal degradation of the citrate carrier Slc25a1. Restoring histone acetylation to youthful levels rescues osteogenesis.

## Main

Stem cell exhaustion is a well-established hallmark of the aging process^1^. Bone marrow mesenchymal stem cells (MSCs) have been shown to play an important role in aging due to their ability to regenerate bone by giving rise to adipocytes, chondrocytes and osteoblasts^2–4^. However, aged MSCs show decreased capacity to differentiate into osteogenic and chondrogenic lineages. This feature has been linked to increased fat content in the bone marrow upon aging, and concomitantly higher risk of osteoporosis and fractures^5,6^.

Chromatin architecture influences stem cell fate decisions, and cell differentiation is often accompanied by chromatin rearrangements^7^. Research over the last few years has shown that aging is accompanied by changes in the chromatin landscape and subsequently by alterations in the gene expression profile across cell types and tissues^8,9^. Recently, partial reprogramming in various tissues was found to promote tissue regeneration and to prolong lifespan in premature aging models^10^, strongly suggesting that interfering with epigenetic mechanisms is a powerful tool to intervene in the aging process.

In this context, it is important to highlight that metabolism and chromatin are heavily intertwined^11–14^. More precisely, intracellular metabolism provides metabolites that serve as essential cofactors and substrates for chromatin-modifying enzymes and their availability can strongly affect the activity of these enzymes. Many of these metabolites are generated in mitochondria and this establishes a tight mitochondrial–nuclear connection^15^. For instance, during histone acetylation, histone acetyl-transferases (HATs) transfer acetyl-groups from acetyl-CoA to lysine residues of histones. Importantly, acetyl-CoA levels directly affect the levels of histone acetylation, independently of the HAT enzymatic activity^16–19^, due to the fact that the Michaelis constant (*K*_m_) of most HATs falls within the range of the cellular acetyl-CoA concentration^20^.

Here, we study the interplay between metabolism and epigenome during aging using bone-marrow-derived MSCs.

## Results

### Chromatin compaction and histone hypoacetylation in old MSCs

**Fig. 1:**
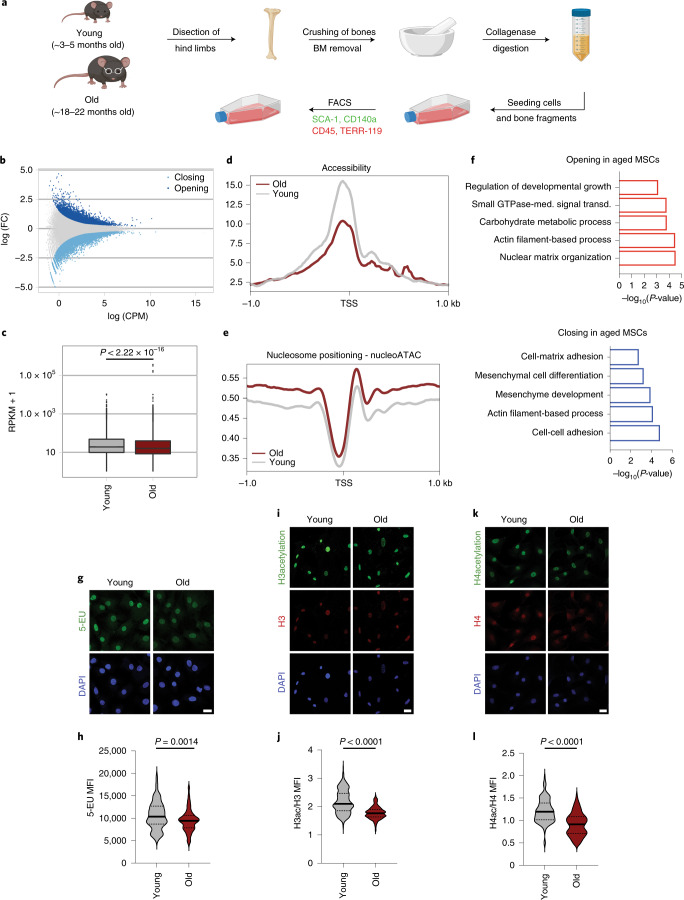
Chromatin compaction and histone hypoacetylation in aged MSCs. **a**, Scheme of the isolation protocol of bone marrow MSCs from the back limbs of young (~3–5 months old) and old (~18–22 months old) mice. **b**, Microarray (MA)-plot showing opening and closing peaks with age, as determined by ATAC-seq, averaged from *n* = 2 biologically independent experiments. **c**, Overall genome accessibility expressed as RPKM values measured by ATAC-seq. Statistical significance was determined by two-sided Wilcoxon test. The *y* axis is scaled to log_10_ for visualization purposes. The lower and upper hinges of the boxplot correspond to the first and third quartiles (25th and 75th percentiles) while the middle line is median and the whiskers extend to 1.5 × interquartile range (IQR) from both lower and upper hinges. The notches extend 1.58 × IQR/sqrt(n), which is roughly 95% confidence intervals (CIs) for comparing medians; *n* = 2 of biologically independent experiments. **d**, Metaplot of ATAC-seq reads over the TSS of all protein-coding genes; *n* = 2 biologically independent experiments. **e**, NucleoATAC metaplot to map position of all nucleosomes around the TSS of all protein-coding genes. **f**, Representation of GO terms analyzed with Metascape^69^ for opening and closing peaks upon aging, identified by ATAC-seq. GTPase-med. signal transd., GTPase-mediated signal transduction **g**,**h**, Representative images (**g**) and quantification (**h**) of mean fluorescence intensity (MFI), after labeling *n* = 140 young cells and *n* = 118 aged cells with 1 mM 5-EU, for 7 h. Nuclei were stained with DAPI. Merged results from *n* = 3 biologically independent samples are shown. **i**–**l**, Representative images (**i**,**k**) and quantification of mean fluorescence intensity (MFI) after immunostaining against H3ac and H3 of *n* = 203 young cells and *n* = 183 aged cells, and against H4ac and H4 of *n* = 115 young cells and *n* = 107 aged cells, respectively. MFI of histone H3 and histone H4 was used as internal control, respectively, for normalization. Nuclei were stained with DAPI; *n* = 3 biologically independent samples. Representative results from one replicate are shown in **j** and **l**. Scale bars (**g**, **i** and **k**), 25 μm. Distribution of data points in **h**, **j** and **l** is shown as violin plots, where the mean is indicated by a solid line and the quartiles are indicated with dashed lines. Statistical significance was determined in (**h**, **j** and **l**) using two-sided unpaired *t*-test.

We isolated MSCs from the endosteum of young and old mice, following a published protocol outlined in Fig. 1a^21^. Since only 500–1,000 MSCs could be isolated per mouse following this procedure, we modified the protocol by adding the possibility for outgrowth of more MSCs from bones in vitro, before cell sorting, to acquire sufficient cells for biochemical assays (~40,000 cells per mouse). Taking into consideration the variability between individual mice, we pooled together cells obtained from three different animals in each biological replicate. To maintain an oxygen concentration similar to that in their niche, MSCs were cultured exclusively under 2% oxygen. For all experiments, cells were kept in culture for a maximum of four to five passages (~4 weeks after cell isolation).

**Extended Data Fig. 1:**
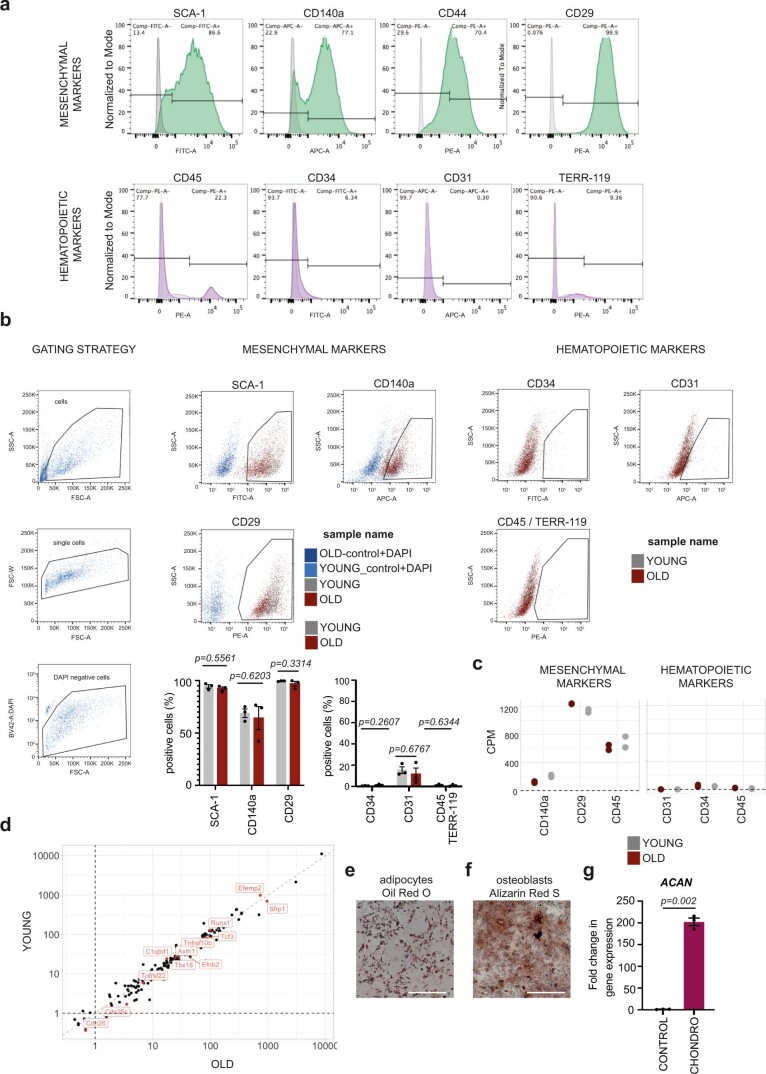
Characterization of young and aged isolated MSCs. **(a-b**) Marker profile of MSCs for mesenchymal and hematopoietic genes, as assessed by flow cytometry, using cells at passage 2 after isolation *(a)* and passage 5 (~4 weeks in culture – *b*). For the bar graphs in *(b)*, data are presented as mean ± SEM. n = 3 biologically independent experiments. **(c)** Validation of results shown in (a) by RNA-seq. Plotted here are the counts per million (CPM) values for the gene in n = 2 independent biological replicate. **(d)** Correlation of the transcription levels of known MSC-specific markers^22^ in young and aged MSCs, using our RNA-seq dataset. n = 2 biologically independent experiments where the values are counts per million and both the axis are scaled to log10. **(e-f)** Multilineage differentiation capacity of MSCs. Representative images *(e and f)* of n = 3 biologically independent experiments, showing adipogenic and osteogenic differentiation, which were confirmed after Oil Red-O and Alizarin Red S staining, respectively. Images were acquired by bright-field microscopy, 12 days after induction of differentiation. Scale bars, 500um. **(g)** Chondrogenic differentiation was confirmed by qPCR analysis of the chondrogenic gene *Acan*, 14 days after induction of differentiation, in comparison to non-induced cells. β-actin was used as an internal control for normalization. n = 3 biologically independent experiments are shown and data are presented as mean ± SEM. Statistical significance in *(b)* and *(g)* was determined using two-sided unpaired t-Test.

Purified MSCs were devoid of hematopoietic markers and showed expression of MSC surface markers (Extended Data Fig. 1a). Importantly, cells did not lose their characteristic cell surface marker identity even after 4 weeks in cell culture, with most cells expressing the SCA-1, CD140a and CD29 mesenchymal markers and being depleted for the CD34, CD31, TERR-119 and CD45 hematopoietic markers, as evidenced by flow cytometry analysis (Extended Data Fig. 1b) and confirmed by RNA-sequencing analysis (RNA-seq; Extended Data Fig. 1c). In line with this, correlation of the transcription levels of known MSC-specific markers^22^ between young and old MSCs showed that the MSC-specific genes were indeed expressed in the isolated MSC population (Extended Data Fig. 1d). Of note, we did not find any difference between young and old cells in the expression levels of these MSC marker genes, indicating that cells from both age groups maintained their mesenchymal transcriptional identity during cell culture. Furthermore, the isolated MSCs possessed trilineage differentiation potential, showing adipogenic, osteogenic and chondrogenic differentiation capacity (Extended Data Fig. 1e–g).

**Extended Data Fig. 2:**
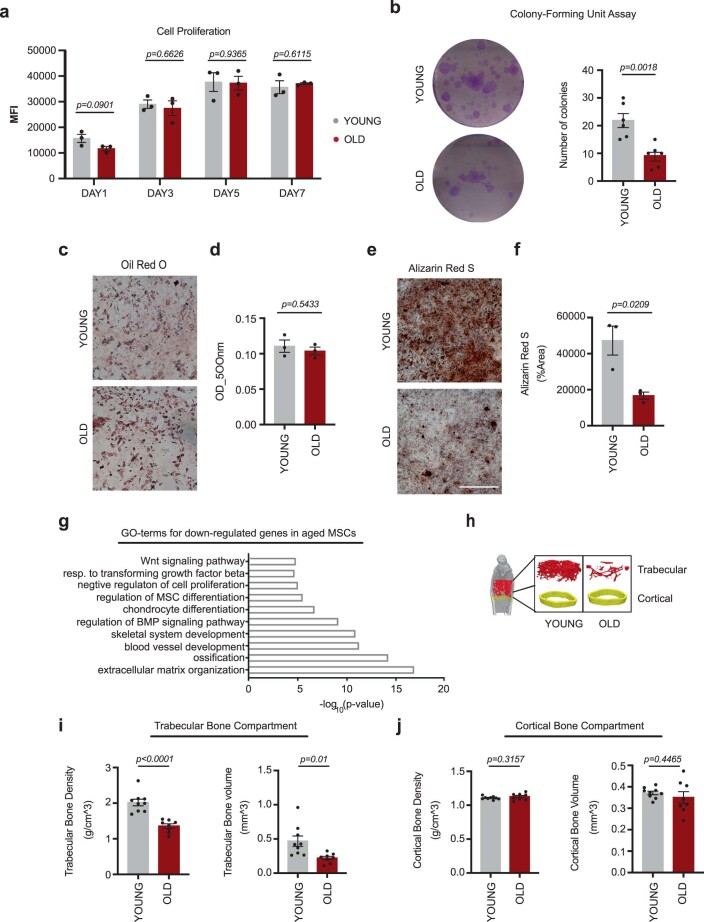
MSCs lose their osteogenic capacity upon aging. **(a)** Comparison of the proliferation rate of young and aged MSCs, after 1,3,5, and 7 days in culture, using the CyQuANT NF Cell Proliferation Assay kit. Results of n = 3 biologically independent experiments are shown and data are presented as mean ± SEM. **(b)** Representative images and quantification of Colony-Forming Unit assay for young and aged MSCs, after culturing them for 10-14 days. Results of n = 6 biologically independent experiments are shown and data are presented as mean ± SEM. **(c-d)** Representative images *(c)* and quantification *(d)* after Oil Red-O staining of young and aged cells, 9 days after induction of adipogenesis. Results of n = 3 biologically independent experiments are shown and data are presented as mean ± SEM. Scale bars, 500um. **(e-f)** Representative images *(e)* and quantification *(f)* of Alizarin Red S staining of young and aged MSCs, 12 days after osteogenesis induction. Images were acquired using bright-field microscopy. Scale bars, 500um. Results of n = 3 biologically independent experiments are shown and data are presented as mean ± SEM. **(g)** GO-term analysis for down-regulated genes upon ageing, identified by RNA-seq. n = 3 biologically independent replicates. **(h-j)** Measurement of bone density and bone volume of the cortical and trabecular bone compartments from the femurs of young and old animals; Results of n = 9 biologically independent experiments of young mice and n = 8 biological replicates for old mice are shown and data are presented as mean ± SEM. Statistical significance in *(a), (b), (d), (f), (i)* and *(j)* was determined using two-sided unpaired t-Test.

MSCs isolated from young and old mice did not differ in their proliferation rate (Extended Data Fig. 2a). However, we observed that cells isolated from old mice exhibited lower proliferation within colonies (Extended Data Fig. 2b) and skewed adipogenic differentiation at the expense of osteogenesis (Extended Data Figs. 2c–f), which are both well-described characteristics of aged MSCs. The decreased proliferation and osteogenesis were in line with the age-dependent changes in the transcriptome profile (Extended Data Fig. 2g). Together, these data demonstrate that our purification and culture strategy enabled successful isolation of pure, multipotent MSCs that maintained their typical characteristics in vitro throughout the time kept in culture. Analysis of several bone quality-related parameters in the young and old donor mice showed a strong decrease in the bone quality of old mice, particularly in the trabecular bone compartment where MSCs reside (Extended Data Fig. 2h–j), mirroring our in vitro findings of lower osteogenesis upon aging.

**Extended Data Fig. 3:**
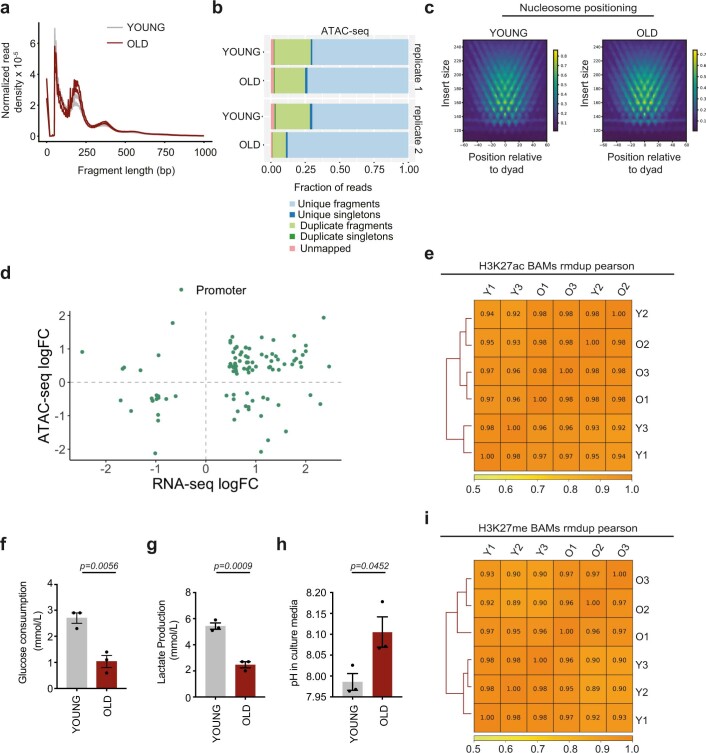
Quality control measurements for the epigenetic analyses. **(a-b)** Insert size distribution of each single ATAC-seq library (2 libraries per age group- *a*) and mapping statistics for each individual replicate *(b)*. **(c)** V-plot to characterize dyad position within the nucleosomes. (d) Correlation of ATAC-seq and RNA-seq data, where the log2 fold change (logFC) of genes differentially expressed in young and aged MSCs and of their differentially accessible promoters is plotted. Only the significantly differentially expressed genes (adjusted p-value <= 0.05) after multiple testing correction were taken into account here. (e) Correlation matrix of three young (Y) and three old (O) replicates, after H3K27ac CUT&RUN-seq. (f-h) Glucose consumption (f), lactate production *(g)* and pH *(h)* measured in the media of young and aged cells using the Vi-Cell MetaFLEX instrument. n = 3 biologically independent replicates. Data are presented as mean ± SEM. Statistical significance was determined using two-sided unpaired t-Test.

Using this system, we sought to investigate the underlying epigenetic determinants of the decreased osteogenic differentiation upon aging. We measured global chromatin accessibility by assay for transposase-accessible chromatin using sequencing (ATAC-seq; Extended Data Fig. 3a,b)^23^ on young and aged MSCs and found that, upon aging, ~6,500 sites in the genome became more accessible, while ~8,250 sites became less accessible (Fig. 1b). Surprisingly, we found that global chromatin accessibility decreased significantly with age (Fig. 1c), opposite to what has been proposed for various organisms^24^.

Given that chromatin accessibility on gene promoters correlates with gene expression, we further analyzed differences in the chromatin structure and plotted accessibility over the transcription start site (TSS), as a metaplot (Fig. 1d). We observed that gene promoters of aged cells exhibited a strong decrease in chromatin accessibility. To validate this result, we used NucleoATAC—an algorithm that allows the precise mapping of nucleosomes based on ATAC-seq datasets^25^. Plotting nucleosome occupancy over the TSS of all protein-coding genes confirmed that aging led to higher nucleosome density at the promoter region (Fig. 1e). A main feature of the aging epigenome is increased nucleosome fuzziness^24^. However, in our aged MSCs, the position of nucleosomes was properly defined (Extended Data Fig. 3c), suggesting that only the abundance of nucleosomes, and not their position, changed upon aging. We next investigated which genes changed promoter accessibility upon aging. Gene ontology (GO) enrichment analysis for genes with reduced promoter accessibility in aged MSCs showed terms surrounding mesenchymal cell differentiation, signaling and cell-matrix adhesion processes (Fig. 1f), in line with the transcriptional changes observed by RNA-seq (Extended Data Fig. 2g).

Chromatin accessibility is linked tightly to the transcriptional output. Therefore, we plotted all significantly changed transcripts upon aging and the respective change on their promoter chromatin architecture. As expected, the change in the transcriptional output correlated strongly with the change in promoter accessibility (Extended Data Fig. 3d). Such a strong decrease in chromatin accessibility should in general lead to a decrease in overall transcriptional output. To address this further, we performed metabolic labeling using 5-ethynyl uridine (5-EU). Consistent with the age-associated loss of chromatin accessibility, aged MSCs showed significantly reduced transcriptional rate compared with young MSCs (Fig. 1g,h). To further investigate the molecular underpinnings for the age-associated chromatin compaction, we analyzed histone acetylation levels. Histone acetylation neutralizes the positive charge of the side-chain of histones and weakens the contact between histones and DNA^26^. Thus, it plays a fundamental role in the regulation of chromatin accessibility. Therefore, we investigated if alterations in the histone acetylation profile contributed to changes in chromatin accessibility. Indeed, we observed significantly decreased levels of total histone H3 and histone H4 acetylation in MSCs from old mice (Fig. 1i–l), suggesting that the loss of chromatin accessibility was largely influenced on the level of histone post-translational modifications.

Taken together, our ATAC-seq data and immunofluorescence results suggest that aging is accompanied by histone hypoacetylation and decreased chromatin accessibility leading to transcriptional changes that are responsible for the lower proliferation and differentiation capacity of aged MSCs.

### Aging redistributes histone marks on lineage-defining genes

**Fig. 2:**
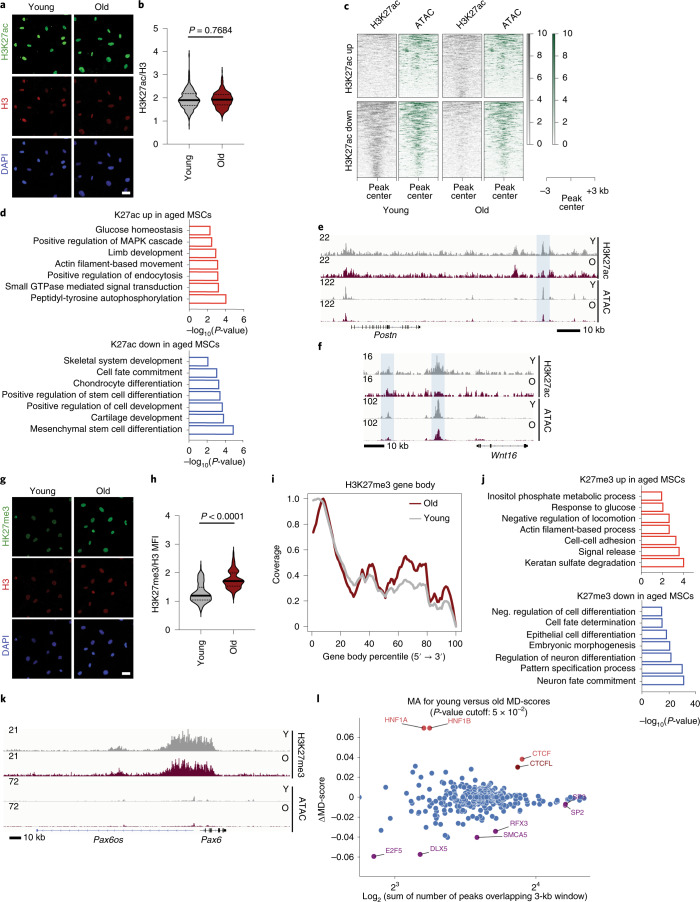
Altered abundance of histone marks on lineage-specific genes in aged MSCs. **a**,**b**, Representative images (**a**) and quantification of MFI after immunostaining against H3K27ac and H3 of *n* = 488 young cells and *n* = 366 aged cells (**b**). MFI of histone H3 was used as an internal control for normalization. Nuclei were stained with DAPI. Merged results from *n* = 3 biologically independent experiments are shown. **c**, Heatmaps of regions overlapping in H3K27ac and ATAC-seq representing potential enhancers. Plots were aligned to the peak center, ±3 kb. **d**, Representation of GO terms analyzed with Metascape^69^ for gene promoters gaining and losing H3K27ac abundance upon aging. **e**,**f**, Integrative genomics viewer (IGV) browser views showing H3K27ac read density (combined from *n* = 3 biologically independent experiments) and ATAC-seq (combined from *n* = 2 biologically independent experiments) in young and aged MSCs, near the *Postn* (**e**) and *Wnt16* (**f**) genes. Shaded regions demonstrate differences between young and aged MSCs. **g**,**h**, Representative images (**g**) and quantification (**h**) of MFI after immunostaining against H3K27me3 and histone H3 of *n* = 372 young cells and *n* = 378 aged cells. MFI of histone H3 was used as an internal control for normalization. Nuclei were stained with DAPI. Merged results from *n* = 3 biologically independent experiments are shown. **i**, H3K27me3 enrichment density (combined from *n* = 3 biologically independent experiments) over the gene body of all protein-coding genes. **j**, GO-term analysis using the Metascape algorithm for regions gaining and losing H3K27me3 abundance upon aging. **k**, IGV browser view showing H3K27me3 read density (combined from *n* = 3 biologically independent experiments) and ATAC-seq (combined from *n* = 2 biologically independent experiments) in young and aged MSCs, near the *Pax6* gene. **l**, DAStk analysis on the ATAC-seq dataset to predict transcription factor activity. Scale bars (**a**,**g**), 25 μm. Distribution of data points in **b** and **h** is shown as violin plots, where the mean is indicated by a solid line and the quartiles are indicated with dashed lines. Statistical significance in **b** and **h** was determined using two-sided unpaired *t*-test.

To understand if aging was also accompanied by changes in other functional elements on the epigenome, we analyzed H3K27 acetylation (H3K27ac) levels, a mark associated with active enhancers. Global levels of H3K27ac were not changed in aged MSCs (Fig. 2a,b). However, to identify whether specific genomic loci showed altered H3K27ac abundance upon aging, we performed CUT&RUN-seq using a H3K27ac-specific antibody (Fig. 2c and Extended Data Fig. 3e). We called putative enhancers by overlapping H3K27ac with ATAC peaks in young MSCs and visualized those as heatmaps (Fig. 2c). Subsequently, we annotated genes on the basis of the ‘closest distance’ method^27–30^. Strikingly, potential enhancers losing H3K27ac abundance, and thus activity, upon aging, were associated with genes encoding for transcripts involved in MSC function, particularly in differentiation into chondrocytes and skeletal system development (Fig. 2d). This indicates that loss of enhancer marking by H3K27ac played a crucial role in the loss of differentiation potential during aging. Examples of genes that lost chromatin accessibility and H3K27ac abundance on potential enhancers upon aging are shown for *Postn* (Fig. 2e) and *Wnt16* (Fig. 2f), which are involved in osteogenesis and stem cell proliferation, respectively. On the other hand, regions that gained H3K27ac abundance upon aging (Fig. 2c) were not associated with lineage-specific terms, but were linked to genes encoding for transcripts involved mainly in glucose homeostasis and signaling (Fig. 2d). To validate this finding, we investigated changes in glucose metabolism between young and aged MSCs. Interestingly, we found that aged MSCs showed lower levels of glucose uptake and lactate secretion (Extended Data Figs. 3f–h), indicating decreased glycolytic activity.

Due to the age-dependent alterations in H3K27ac and the increase in chromatin compaction, we wondered whether trimethylation of H3K27 (H3K27me3) was altered too, as this modification is associated with gene silencing. Indeed, we observed a global increase in H3K27me3 levels (Fig. 2g,h), supporting our previous findings of a chromatin state that is transcriptionally less permissive. To identify the precise genomic regions that showed changes in the H3K27me3 status upon aging, we performed CUT&RUN-seq using a H3K27me3-specific antibody (Extended Data Fig. 3i). While H3K27me3 abundance close to gene promoters did not change between young and aged MSCs, we observed an increase in H3K27me3 abundance across the gene body (Fig. 2i). Notably, genes gaining H3K27me3 were associated with GO terms enriched for metabolic processes and cell adhesion terms, whereas genes identified to lose H3K27me3 were strongly enriched for terms involved in neuronal differentiation (Fig. 2j). However, the loss of H3K27me3 on these genes was only mild, as shown for the *Pax6* gene, which regulates development of neural tissues (Fig. 2k), and the ATAC-seq tracks did not show any increase in promoter accessibility. Thus, silencing of these genes was probably not strongly altered with age.

Finally, we used our ATAC-seq analysis to interrogate if specific transcription factors were responsible for the observed chromatin rearrangements upon aging. To answer this question, we applied the Differential ATAC-seq toolkit (DAStk)^31^, which predicts transcription factor activity, to our ATAC-seq data. DAStk analysis identified several transcription factors and chromatin remodelers that could have contributed to the accessibility changes observed by ATAC-seq (Fig. 2l). The most significant hit was CTCF, which was predicted to be more active in aged cells. Interestingly, CTCF is known to regulate chromatin architecture, and, thus, gene transcription. By contrast, higher activity of the E2F5 and DLX5 transcription factors was implicated in young cells. These transcription factors are involved in cell proliferation and osteogenesis, respectively, and their predicted alterations supported the observed changes on lineage-specific enhancers and the age-related defects on proliferation and osteogenesis capacity (Extended Data Fig. 2b–j).

In sum, in our system, aging is not only accompanied by global changes in chromatin accessibility, but also involves lineage-specific alterations to functional elements in the genome, which explain the impaired osteogenic phenotype observed in aged MSCs.

### Acetyl-CoA accumulates in aged MSCs due to low lipogenesis

**Fig. 3:**
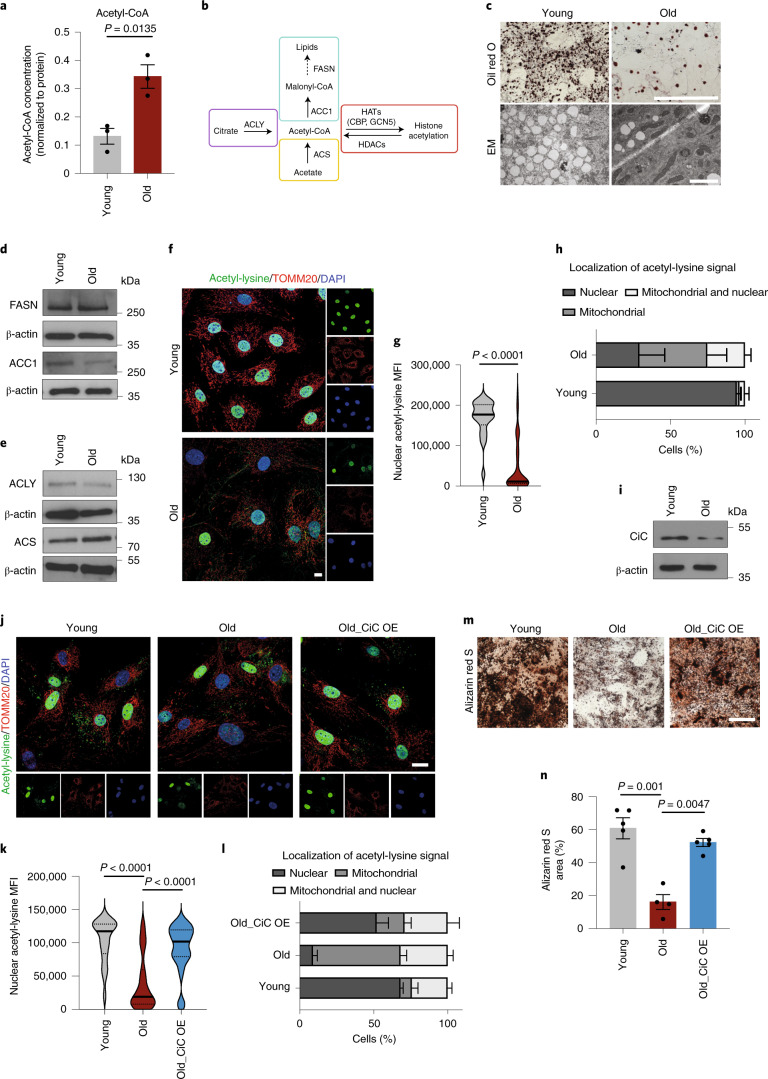
Age-associated loss of histone acetylation and osteogenesis due to lower CiC levels. **a** Measurement of acetyl-CoA using Mass Spectrometry analysis. n = *3* biologically independent replicates. **b** Schematic graph describing the different metabolic pathways generating and consuming acetyl-CoA and the main associated enzymes. **c**, Representative images of lipid droplets. Images were acquired by bright-field microscopy after Oil red O staining (top) and by electron microscopy (EM, bottom). Scale bars, 500 μm for bright-field microscopy images and 2 μm for EM images; *n* = 3 biologically independent replicates. **d**,**e**, Representative immunoblots for FASN and ACC1 (**d**), ACLY and ACS (**e**) proteins. β-actin, loading control; *n* = 3 biologically independent replicates. **f**–**h**, Representative images after immunostaining against acetyl-lysine and TOMM20 (**f**), quantification of nuclear acetyl-lysine MFI in *n* = 47 young cells and *n* = 67 aged cells (**g**) acetyl-lysine signal localization after manual assignment into three categories: exclusively nuclear, exclusively mitochondrial and nuclear/mitochondrial (**h**). Nuclei were stained with DAPI. Merged results from *n* = 3 biologically independent young replicates (*n* = 264 cells) and *n* = 2 biologically independent aged replicates (*n* = 119 cells) are shown in **h**. Scale bars, 10 μm. **i**, Representative immunoblot for CiC. β-actin, loading control. *n* = 3 biologically independent replicates. **j**–**l**, Representative images after immunostaining against acetyl-lysine and TOMM20 (**j**), quantification of nuclear acetyl-lysine MFI in *n* = 41 young cells, *n* = 37 aged cells and *n* = 33 CiC-over-expressing (CiC_OE) aged cells (**k**) and acetyl-lysine signal localization, as described in **h** and **l**. Nuclei were stained with DAPI. *n* = 3 biologically independent replicates; results of one representative biological replicate are shown in **k**, whereas merged results of the *n* = 3 biological replicates (*n* = 123 young cells, *n* = 104 aged cells and *n* = 122 CiC-transfected aged cells are shown in **l**. Scale bars, 25 μm. **m**,**n**, Representative images and quantification after Alizarin red S staining. Images were acquired using bright-field microscopy. Quantification of staining was done in ImageJ, quantifying *n* = 5 randomly selected fields in young and *n* = 4 randomly selected fields in Old and Old_CiC OE samples. *n* = 3 biologically independent samples and results from one representative replicate are shown in **n**. Scale bars, 500 μm. For bar graphs, data are presented as mean ± s.e.m. In **g** and **k**, distribution of data points is shown as violin plots, where the mean is indicated by a solid line and the quartiles are indicated with dashed lines. Statistical significance was determined in **a**, **g**, **h**, **k**, **l** and **n** using two-sided unpaired *t*-test.

**Extended Data Fig. 4:**
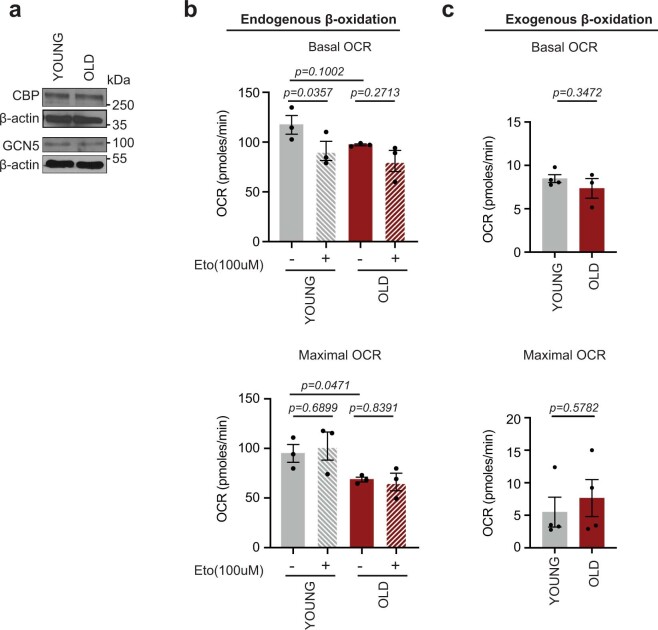
Metabolic alterations during MSC aging. **(a**) Representative immunoblots for CBP and GCN5 histone acetyl-transferases in young and aged MSCs. β-actin, loading control. n = 2 biologically independent replicates. **(b)** Basal (top) and maximal (bottom) Oxygen Consumption Rates (OCR) in young and aged MSCs, treated with or without 100 μM Etomoxir for 1 hour, prior to the assay. Measurements were made using the SeaHorse XF24 extracellular flux analyzer instrument. n = 3 biologically independent replicates. **(c)** Basal (top) and maximal (bottom) OCR in young and aged MSCs. Cells were incubated overnight with substrate-limited medium. On the day of the assay, BSA-Palmitate was injected to cells. Measurements were made using the SeaHorse XF24 extracellular flux analyzer instrument. n = 4 biologically independent replicates in young and n = 3 biologically independent replicates in old. Data in *(b)* and *(c)* are presented as mean ± SEM. Statistical significance was determined using two-sided unpaired t-Test.

Our findings prompted us to investigate how the histone acetylation profile was established in young and aged MSCs and whether other cellular processes were involved in this phenotype. Of note, the levels of the two main HATs CBP and GCN5 did not decrease upon aging (Extended Data Fig. 4a), suggesting that the loss of histone acetylation in aged cells was not due to differential HAT expression. Thus, we next focused on the metabolism-produced acetyl-CoA, because its local availability directly influences levels of histone acetylation, independently of the enzymatic activity of HATs^32,33^. Unexpectedly, we found increased acetyl-CoA levels in aged MSCs (Fig. 3a). This result was in contradiction to the histone hypoacetylation observed in MSCs from old mice (Fig. 1i–l) and motivated us to investigate further where the higher levels of acetyl-CoA originated from and what they were used for.

One main metabolic pathway that heavily generates and consumes acetyl-CoA is lipid metabolism (Fig. 3b). To understand if lipid content changes with age, we stained cells with Oil red O to visualize neutral lipids. We found that aged MSCs had much weaker lipid staining, indicating lower content of neutral lipids (Fig. 3c). This observation was also confirmed with electron microscopy data that showed a strong decrease in the amount of lipid droplets (LDs) in aged MSCs (Fig. 3c). The lower LD content could be explained both by lower fatty acid biosynthesis and increased lipid consumption through β-oxidation. Focusing on potential alterations in β-oxidation upon aging, we initially analyzed changes in lipid metabolism after treating cells with etomoxir, which inhibits endogenous β-oxidation, and after supplementation with palmitate, which is the main energy source in starved cells. Etomoxir treatment did not affect mitochondrial activity in aged MSCs (Extended Data Fig. 4b), suggesting that these cells did not rely heavily on endogenous fatty acid oxidation to produce energy. Furthermore, comparison of mitochondrial respiration after palmitate supplementation showed that there was no significant change between young and aged MSCs (Extended Data Fig. 4c), indicating that, in aged MSC, there was no higher activation of the exogenous fatty acid oxidation pathway. Together, these data suggest that the higher acetyl-CoA levels, and particularly the lower lipid content of aged MSCs, were not due to increased lipid consumption through β-oxidation. Therefore, we investigated changes in the lipid biogenesis pathway and compared the levels of key enzymes involved in de novo synthesis of fatty acids. Although FASN protein levels did not change upon aging, levels of the ACC1 enzyme, which is important for the initiation of the fatty acid biosynthesis pathway, decreased strongly in aged cells (Fig. 3d). In agreement with our previous finding of lower LDs content in aged cells (Fig. 3c), this result suggests that acetyl-CoA was not used to generate de novo fatty acids; thus, it accumulated in the aged MSCs.

### Acetyl-CoA trapping inside mitochondria leads to chromatin compaction and impairs osteogenesis

Since aged cells contained higher levels of acetyl-CoA (Fig. 3a), one potential explanation for the reduced lipid biogenesis and histone acetylation could be a decrease in the cytosolic pool of acetyl-CoA. However, levels of the enzymes ATP-citrate lyase (ACLY) and acetyl-CoA synthetase (ACS), which are involved in the cytoplasmic/nuclear generation of acetyl-CoA^32,34^, were stable upon aging (Fig. 3e). Hence, we speculated that aged cells exhibit impaired acetyl-CoA export from mitochondria to the cytoplasm. To test this hypothesis, we took into consideration the fact that mitochondrial proteins are acetylated in a nonenzymatic manner when acetyl-CoA concentration inside mitochondria is high^35,36^. We observed a strong, age-dependent change in the localization of acetyl-lysine signal, shifting from nuclear to mitochondrial upon aging (Fig. 3f–h), indicating that acetyl-CoA was indeed trapped inside mitochondria of aged cells.

**Extended Data Fig. 5:**
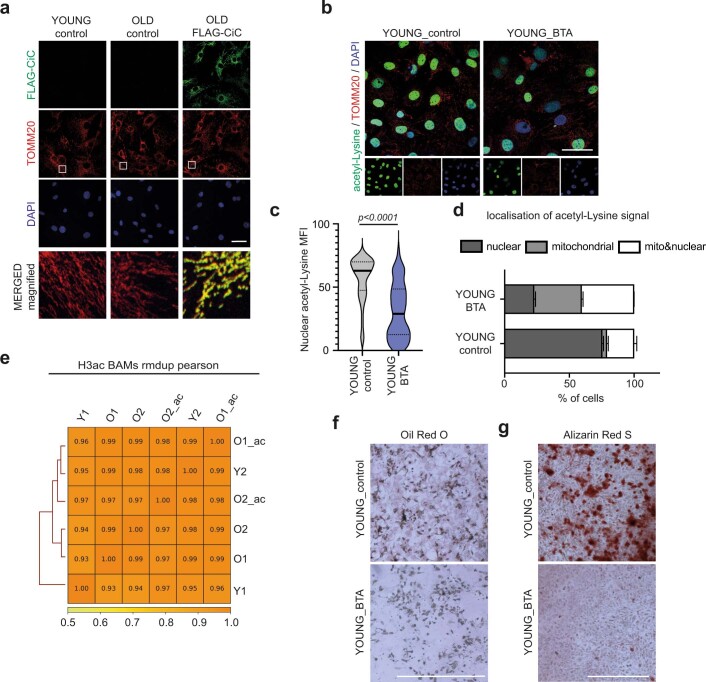
Inhibition of CiC function by BTA impairs osteogenesis. **(a)** Representative images after staining control young and aged MSC and CiC-FLAG- expressing aged MSCs, using an anti-FLAG antibody. TOMM20 was used as a counterstain for mitochondria, to confirm proper localization of the exogenously expressed CiC-FLAG protein, as shown in magnification. Transfection and all downstream experiments were done for n = 3 biologically independent replicates. Scale bar; 50um. **(b-d)** Representative images after staining of control and BTA-treated young cells with acetyl-Lysine and TOMM20 antibodies *(b)*, quantification of nuclear acetyl-Lysine mean fluorescence intensity (MFI) in control (n = *62*) and BTA-treated (n = *57*) cells *(c)* and assessment of acetyl-Lysine signal localization after manual assignment into three categories: exclusively nuclear, exclusively mitochondrial and nuclear/mitochondrial *(d)*. Nuclei were stained with DAPI. Merged results from n = 2 biologically independent experiments of control (n = *162 cells*) and BTA-treated (n = *115 cells*) samples are shown in (*d*). Scale bars; 50um. **(e)** Correlation matrix of n = 2 young (Y), n = 2 old (O) and n = 2 acetate-treated old (O_ac) biologically independent replicates, after H3ac CUT&RUN-seq. **(f)** Representative images after Oil Red-O staining of young cells treated with or without 1 mM BTA for 3 days before induction of adipogenesis. Images were acquired using bright-field microscope. Scale bar, 1 mm; n = 3 biological replicates. **(g)** Representative images of Alizarin Red S staining performed on young MSCs, 8 days after induction of osteogenesis. Cells were treated with or without 1 mM BTA for 3 days before induction of osteogenesis. Images were acquired using bright-field microscope. Scale bar, 1 mm. n = 3 biologically independent replicates. Distribution of data points in *(c)* is shown as a violin plot, where the mean is indicated by a solid line and the quartiles are indicated with dashed lines. Statistical significance in *(c)* and *(d)* was determined using unpaired two-sided t-Test. p-values for *(d)* are 0.0248 (for nuclear), 0.0019 (for mitochondrial) and 0.0701 (for mito&nuclear).

To explain why aged cells showed such a compartmentalized localization of acetyl-CoA, we focused on CiC, which exports acetyl-CoA from mitochondria in the form of citrate^37^. Cytosolic citrate is then is converted into acetyl-CoA by ACLY enzyme (Fig. 3b). Importantly, a connection between CiC and histone acetylation has been demonstrated in *Drosophila melanogaster* and in primary human fibroblasts^38^. Surprisingly, CiC protein levels decreased strongly in aged cells (Fig. 3i), indicating that MSCs from old mice showed impaired export of acetyl-CoA from mitochondria to the cytosol. To critically test whether CiC was indeed the mechanistic target linking mitochondrial acetyl-CoA to histone acetylation in aged cells, we expressed the FLAG-tagged-CiC exogenously in aged cells, using lentiviral transduction. Overexpressed CiC localized properly to mitochondria (Extended Data Fig. 5a). Elevated CiC levels were sufficient to rescue acetyl-CoA export and histone acetylation in aged MSCs (Fig. 3j–l). Next, we sought to investigate the functional consequences of the CiC-mediated mitochondrial–nuclear communication. Since efficient differentiation of MSCs towards adipocytes and osteoblasts requires oxidative metabolism and a fully functional lipid biosynthesis pathway^32^, we speculated that the decrease of CiC levels observed in the aged cells might contribute to the lower osteogenic differentiation potential of aged MSCs. Indeed, exogenous CiC expression in aged MSCs significantly improved their osteogenic capacity (Fig. 3m,n), confirming our hypothesis that CiC was responsible for the maintenance of MSC activity upon aging.

Taken together, impaired export of acetyl-CoA from mitochondria due to decreased levels of CiC underlies the observed age-dependent chromatin compaction and negatively affects the osteogenic differentiation capacity. Strikingly, restoring CiC levels and re-establishing chromatin plasticity is sufficient to rejuvenate aged MSCs with respect to their osteogenic potential.

### Restoring histone acetylation rescues aging-driven osteogenic defects

**Fig. 4:**
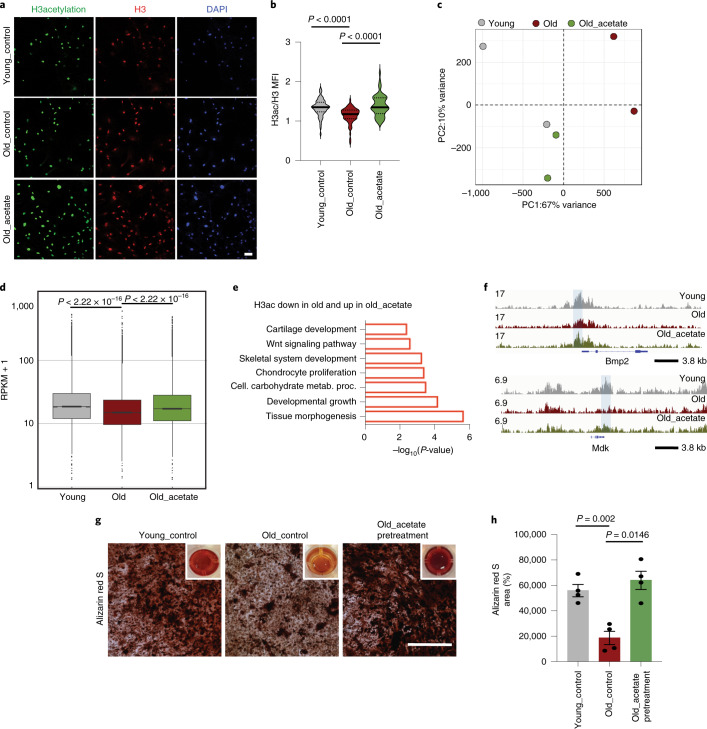
Sodium acetate remodels chromatin and improves the osteogenic capacity of aged MSCs. **a**,**b**, Representative images (**a**) and quantification (**b**) of MFI after immunostaining against histone H3ac and histone H3 of *n* = 144 young cells, *n* = 67 control aged cells and *n* = 53 treated aged cells, treated with 5 mM sodium acetate for 3 days. MFI of histone H3 was used as an internal control, for normalization. Nuclei were stained with DAPI. *n* = 3 biologically independent replicates. Scale bars, 75 μm. **c**, Principal component analysis (PCA) plot showing clustering of young, aged and acetate-treated aged cells, after CUT&RUN-seq using H3ac antibody. *n* = 2 biological replicates. **d**, Boxplot showing the peaks quantified as RPKM values in young, aged and acetate-treated aged cells, after CUT&RUN-seq using H3ac antibody from *n* = 2 independent biological replicates. The statistical significance was determined by two-sided Wilcoxon test for each comparison. The *y* axis is scaled to log_10_ for visualization purposes. The lower and upper hinges of the boxplot correspond to the first and third quartiles (25th and 75th percentile) while the middle line is median and the whiskers extend to 1.5 × IQR from both lower and upper hinges. The notches extend 1.58 × IQR/sqrt(n), which is roughly 95% CIs for comparing medians. **e**, Representation of GO terms analyzed with Metascape^69^ for genes that regained promoter and enhancer H3ac abundance after acetate supplementation. Cell. carbohydrate metab. proc., cellular carbohydrate metabolic process. **f**, IGV browser views showing H3ac read density (combined from *n* = 2 biologically independent experiments) in young, aged and acetate-treated aged MSCs, near *Bmp2* (top) and *Mdk1* (bottom) genes. Shaded regions demonstrate differences in young, old and old_acetate samples. **g**,**h**, Representative images (**g**) and quantification (**h**) after Alizarin red S staining of young and aged MSCs treated with or without 5 mM sodium acetate for 3 days before induction of osteogenesis. Images were acquired using bright-field microscopy. Quantification of staining was done in ImageJ, quantifying *n* = 4 randomly selected fields per sample. *n* = 3 biologically independent samples and results from one representative replicate are shown in **h**. Scale bars, 500 μm. For the bar graph in **h**, data are presented as mean ± s.e.m. In **b**, distribution of data points is shown as violin plots, where the mean is indicated by a solid line and the quartiles are indicated with dashed lines. Statistical significance was determined in **b** and **h** using two-sided unpaired *t*-test.

To further explain the CiC–histone acetylation axis, we used 1,2,3-benzene-tricarboxylic acid (BTA), a specific CiC inhibitor 33^39,40^, to modulate CiC activity in young MSCs and analyzed levels of nuclear–mitochondrial acetyl-lysine signal. Inhibition of CiC activity in young cells decreased levels of nuclear acetylation and redistributed the acetyl-lysine signal inside the mitochondria (Extended Data Fig. 5b–d), mimicking the results observed in aged MSCs. In a complementary approach, we added sodium acetate directly to the medium of aged cells. Sodium acetate is an exogenous source of acetyl-CoA and can be converted into acetyl-CoA in the cytoplasm by the ACS enzyme^41^ (Fig. 3b), whose levels did not change with age (Fig. 3e). Impressively, supplementation of aged cells with sodium acetate rescued the loss of histone acetylation, resulting in levels similar to those of young cells (Fig. 4a,b). Collectively, these data corroborate our observation that loss of CiC upon aging is responsible for the reduction of histone acetylation levels, providing a mechanistic link between mitochondrial metabolism and histone acetylation upon aging.

To gain more insight into the effect of sodium acetate on histone acetylation on a global scale, we performed CUT&RUN-seq on young, old and acetate-treated-old MSCs (Extended Data Fig. 5e). Surprisingly, treatment with acetate was sufficient to restore H3ac levels genome-wide, so that aged cells treated with acetate clustered closer to the young cells than to the untreated cells from the same age group (Fig. 4c,d). GO enrichment analysis for genes associated with promoter and enhancer regions that regained H3ac abundance upon acetate supplementation showed terms surrounding skeletal and cartilage development (Fig. 4e), such as the *Bmp2* and *Mdk1* genes (Fig. 4f). These findings indicate strongly that CiC-mediated export of acetyl-CoA from mitochondria to the cytosol is indispensable for histone acetylation and implicate the importance of chromatin accessibility in the osteogenic potential of MSCs. To confirm the role of the CiC–histone acetylation axis in the regulation of the osteogenic capacity of MSCs, we treated aged cells with sodium acetate for 3 days, before induction of differentiation into osteocytes. Surprisingly, acetate pretreatment restored the capacity of aged cells to give rise to osteoblasts to levels similar to those in young cells (Fig. 4g, h), in agreement with the H3ac CUT&RUN-seq results. On the other hand, we used BTA to treat young MSCs for 3 days, before induction of cell differentiation into adipocytes and osteoblasts. Whereas adipogenesis was affected only mildly by CiC inhibition (Extended Data Fig. 5f), osteogenic differentiation decreased strongly in BTA-pretreated cells (Extended Data Fig. 5g).

Collectively, our data suggest that chromatin accessibility and histone acetylation are linked directly to MSC osteogenic potential via CiC activity. Modulating the availability of cytosolic acetyl-CoA remodels chromatin and directly affects osteogenesis.

### CiC is degraded in the lysosomes of aged MSCs

**Extended Data Fig. 6:**
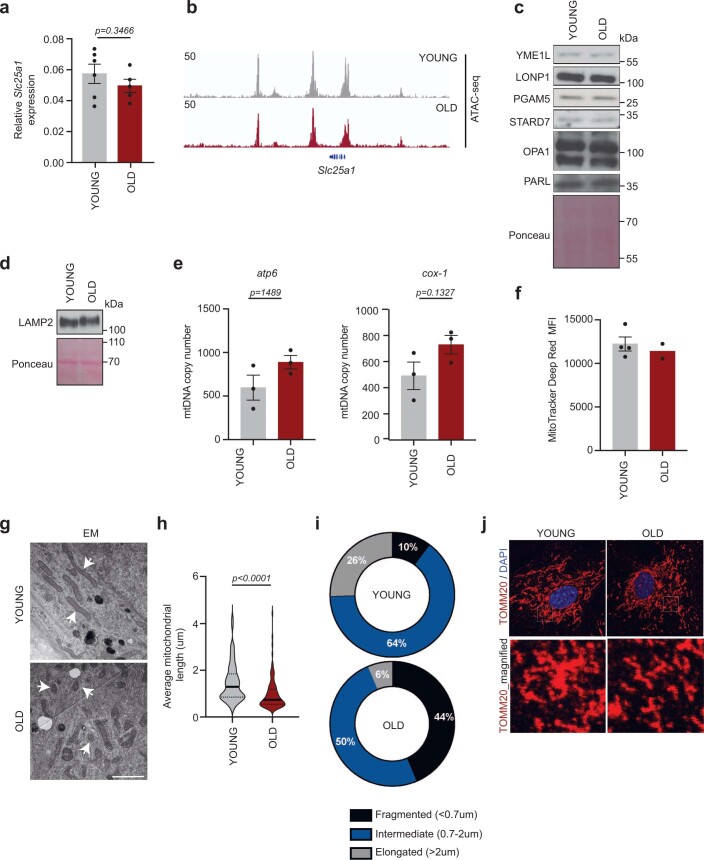
Comparison of diverse mitochondrial parameters between young and aged MSCs. **(a**) qPCR analysis of the expression levels of the *Slc25a1* gene, which encodes the citrate carrier. β-actin expression levels were used as an internal control for normalization. n = 6 biological replicates for young cells and n = 5 biological replicates for aged cells. **(b)** Genome Browser views showing chromatin accessibility assessed by ATAC-seq in young and aged MSCs, near the *Slc25a1* gene. **(c)** Representative immunoblots for YMEL1, LONP1, PGAM5, STARD7, OPA1 and PARL proteins. Membranes were stained with Ponceau S, loading control. n = 3 biologically independent replicates. **(d)** Representative immunoblot for LAMP2 in young and aged MSCs. Ponceau S, loading control. n = 3 biologically independent replicates. **(e)** Quantification of mtDNA content in young and aged cells by qPCR analysis of the expression levels of the mitochondria-encoded a*tp6* and *cox-1* genes. Expression levels of the nucleus-encoded β-actin was used as an internal control, for normalization. n = 3 biologically independent replicates. **(f)** Mean Fluorescence Intensity (MFI) of young and aged cells after staining with MitoTracker Deep Red FM dye. Analysis was performed by flow cytometry; n = 4 biologically independent replicates for young mice and n = 2 biologically independent replicates for old mice. **(g-i)** Observation of mitochondrial structure under the electron microscope *(g)*, analysis of the average mitochondrial length of 60-120 mitochondria from n = 3 biologically independent young and aged *(h)* and manual classification of mitochondria into three categories: fragmented (length<0.7um), intermediate (length:0.7-0.2um) and elongated (length>0.2um); *(i)*. Scale bars, 2um. **(j)** Representative images after TOMM20 staining of young and aged cells. Nuclei were stained with DAPI. Higher magnification of areas within the box is shown in the panel below each image. Scale bars, 25um. n = 3 biologically independent replicates. Data in *(a), (e)* and *(f)* are shown as mean ± SEM. Data in *(h)* is shown as a violin plot, where the mean is indicated by a solid line and the quartiles are indicated with dashed lines. Statistical significance in *(a), (e), (f)* and *(h)* was determined using unpaired two-sided t-Test.

Next, we sought to investigate the mechanism responsible for the reduction of CiC levels in aged MSCs. We first analyzed expression of the *Slc25a1* gene, which encodes the CiC protein. While there was a tendency towards lower *Slc25a1* mRNA in aged MSCs, no significant decrease was observed between young and aged cells (Extended Data Fig. 6a). This finding was also supported by the fact that chromatin features regulating *Slc25a1* expression, such as the chromatin accessibility on *Slc25a1* promoter region, did not change with age (Extended Data Fig. 6b). The absence of transcriptional regulation pointed towards a post-transcriptional and/or post-translational control of CiC levels.

**Fig. 5:**
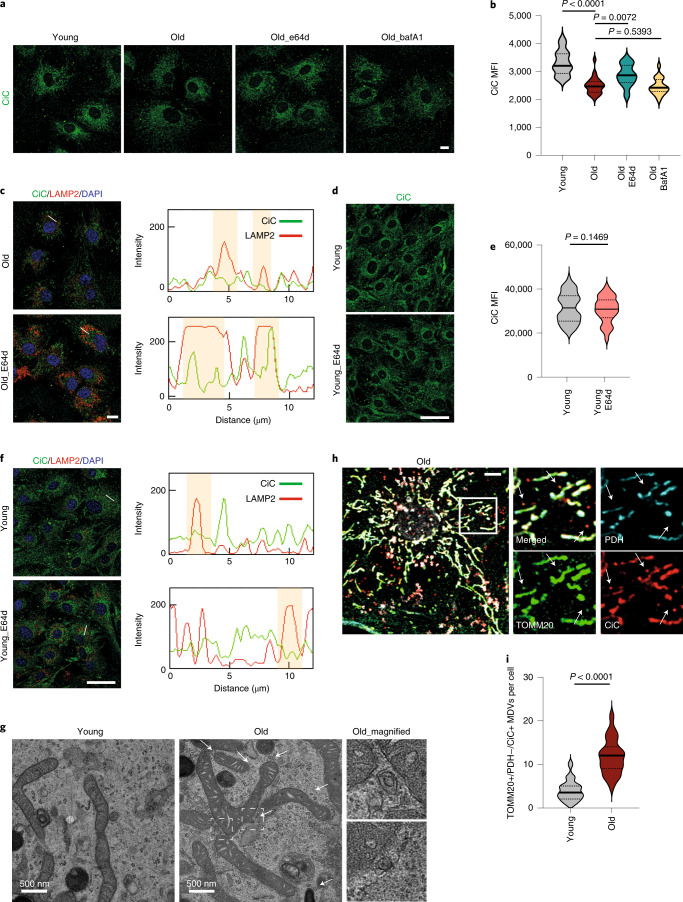
MDV-lysosomal degradation of CiC in aged MSCs. **a**,**b**, Representative images after immunostaining against CiC (**a**) and quantification of MFI of young and aged control cells, following indicating treatments (**b**). Nuclei were stained with DAPI. Scale bar, 10 μm. *n* = 3 biologically independent replicates and 1 representative replicate (*n* = 27 cells for young, *n* = 25 cells for old, *n* = 20 cells for E64d and *n* = 21 cells for BafA1) is shown in **b**. **c**, Representative images after immunostaining of cells from **a** against CiC and LAMP2 (right) and intensity plots of line scans showing the intensity of CiC (green line) and LAMP2 (red line). Shaded regions highlight the CiC signal in areas with increased lysosomal intensity. Nuclei were stained with DAPI. Scale bar, 10 μm. *n* = 3 biologically independent replicates. **d**,**e**, Representative images after immunostaining against CiC (**d**) and quantification of MFI of young control and young E64d-treated cells (**e**). Nuclei were stained with DAPI. Scale bar, 50 μm. *n* = 3 biologically independent replicates and merged results (*n* = 68 cells for young_control and *n* = 80 cells for young_BTA samples) are shown. **f**, Representative images after immunostaining of cells from **d** against CiC and LAMP2 (right) and intensity plots of line scans, as described in **c**. Nuclei were stained with DAPI. Scale bar, 50 μm. *n* = 3 biologically independent samples. **g**, Representative images of MDVs highlighted with arrows, in young and aged cells and higher magnification of the areas in the boxes. Scale bars, 500 nm. *n* = 3 biologically independent samples. **h**,**i**, Representative images after immunostaining against TOMM20, CiC and PDH and quantification of TOMM20+/PDH−/CiC+ MDVs (**h**), shown in higher magnification of areas in the box. *n* = 2 biologically independent replicates and results from 1 representative replicate (*n* = 18 cells for young and *n* = 23 cells for old samples) is shown as violin plot (**i**). Nuclei were stained with DAPI. Scale bar, 8 μm. Distribution of data points in **b**, **e** and **i** is shown as violin plots, where the mean is indicated by a solid line and the quartiles are indicated with dashed lines. Statistical significance in **b**, **e** and **i** was determined using two-sided unpaired *t*-test.

Mitochondrial proteases maintain mitochondrial function and integrity by eliminating unfolded or damaged proteins via degradation. Hence, we initially investigated whether mitochondrial proteases could be involved in the regulation of CiC levels upon aging. We found that levels of several well-known mitochondrial proteases, as well as of their targets^42^, were similar between young and aged MSCs, indicating that their activity did not change with age (Extended Data Fig. 6c) and that they could not be responsible for the regulation of CiC levels. Apart from mitochondrial proteases, two other mitochondrial quality control mechanisms regulate levels of mitochondrial proteins via targeted degradation: autophagy and lysosomal degradation. Therefore, we next investigated if either of these two mechanisms were responsible for the lower CiC levels in aged MSCs. We used Bafilomycin A1 (BafA1), which is a well-known inhibitor of autophagy, and E64d, which blocks lysosomal activity, to treat our cells. We treated aged cells with either vehicle or the indicated drug and analyzed CiC levels. While BafA1 treatment did not increase CiC levels in aged MSCs, treatment with E64d increased the levels of CiC significantly (Fig. 5a,b), indicating that lysosomal activity is important for the degradation of CiC. If this was indeed the case, CiC would be degraded in active lysosomes, whereas it would accumulate in lysosomes after inhibition of their activity by E64d. To test this hypothesis, we co-stained our control and E64d-treated aged cells with CiC and the lysosomal marker LAMP2. Remarkably, levels of LAMP2 remained stable upon aging, arguing that there is no increase in total lysosomal activity and content (Extended Data Fig. 6d). CiC and LAMP2 colocalized to a greater extent in aged, E64d-treated cells, confirming that, in aged MSCs, CiC was degraded in the lysosomes (Fig. 5c). Notably, although inhibition of the lysosomal activity in aged cells restored CiC levels and resulted in accumulation of CiC in the lysosomes, E64d treatment of young cells did not affect CiC levels and localization (Fig. 5d–f), indicating that lysosomal degradation of CiC occurs specifically in aged MSCs.

Lysosomal degradation of proteins residing in the inner mitochondrial membrane was described recently as one potential function of mitochondrial-derived vesicles (MDVs)^43^. Mitochondrial quality control via MDVs occurs in mildly stressed mitochondria, as an initial stress response, to prevent total mitochondrial degradation through mitophagy^44^. To address whether MDVs might play a role in the mitochondrial–lysosomal transport of CiC in aged MSCs, we observed young and aged MSCs under the electron microscope. We identified vesicles showing the described characteristics for MDVs^44^: double-membrane vesicles in close proximity or budding of mitochondria were easily detectable in aged MSCs, while their abundance was much lower in young cells (Fig. 5g). This finding indicates that CiC could indeed be degraded in the aged MSCs via the MDV-lysosomal pathway.

An increase in MDVs also argues for an increase in mitochondrial stress levels in the aged cells—a phenomenon that has been well documented over the last decades^1^. However, stress levels should not exceed the level at which mitophagy would be activated to remove dysfunctional and damaged mitochondria. In line with this hypothesis, we did not observe any large change in mitochondrial DNA (mtDNA) content and membrane potential (Extended Data Fig. 6e,f). By contrast, we observed an increase in mitochondrial fragmentation (Extended Data Fig. 6g–j), which has been linked to mild mitochondrial stress^45,46^. Given that aged cells fulfilled all known criteria for formation of MDVs, which were also observed under the microscope (Fig. 5g), we last sought to validate the cargo selectivity of the MDVs in aging MSCs.

While we still lack a detailed understanding of the mechanisms driving MDV generation and cargo incorporation, MDVs are usually characterized by their proximity to mitochondria, their defined and uniform size of 70–150 nm and the presence or absence of specific mitochondrial membrane proteins. The two main classes of MDVs that have been best characterized so far are TOMM20+/PDH− and TOMM20−/PDH+ MDVs^44^. Hence, we stained aged cells with CiC, TOMM20 and PDH antibodies to investigate whether, in aged MSC, there were MDVs containing CiC. Of note, we did not observe PDH+/TOMM20− MDVs carrying CiC in any of the two age groups. However, we found that aged MSCs showed a strong increase in CiC+/TOMM20+/PDH− MDVs when compared with young cells, indicating that, in aged MSCs, CiC was indeed incorporated exclusively into TOMM20+/PDH− MDVs. The increase in CiC-containing MDVs correlated well with the enhanced lysosomal degradation of CiC, suggesting that CiC levels might indeed be regulated upon aging via MDV-mediated lysosomal degradation (Fig. 5h,i).

### The CiC–histone acetylation axis is conserved in human MSCs

The reduction of the osteogenic potential upon aging is a main contributor to the increased risk of development of osteoporosis with age^47^. To test if the pathway we describe here is also conserved in humans, we used human MSCs derived from the bone marrow upon hemiarthroplasty or total hip arthroplasty^48^ and analyzed CiC and histone acetylation levels. Similar to our observations in murine aged MSCs, CiC levels were decreased in old individuals suffering from osteoporosis, compared with young healthy individuals (Fig. 5a,b). As a consequence of lower CiC levels and decreased cytosolic acetyl-CoA, we also found that histone H3 acetylation levels were lower in the old individuals (Fig. 5c,d). These data suggest that the CiC–histone acetylation link characterized in mouse MSCs is conserved in humans, with an age-dependent decrease in CiC levels resulting in reduced histone acetylation and chromatin plasticity.

## Discussion

**Fig. 6:**
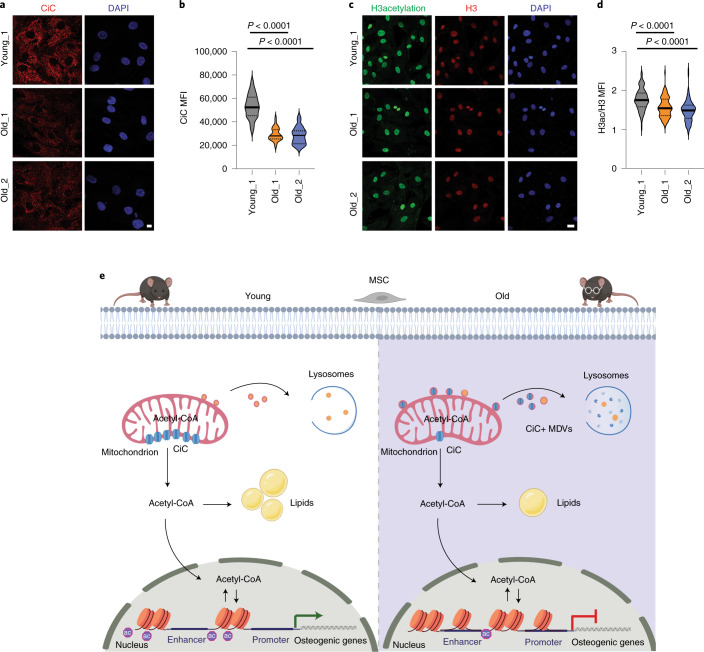
The CiC–histone acetylation-cell fate axis is conserved in human MSCs upon aging. **a**,**b**, Representative images (**a**) and quantification (**b**) of MFI after immunostaining against CiC of young (*n* = 29) and aged (*n* = 48 for old_1 and *n* = 35 for old_2, respectively) cells. Nuclei were stained with DAPI. Scale bars, 10 μm. **c**,**d**, Representative images after immunostaining against H3ac and H3 (**c**) and quantification of MFI of young (*n* = 140 cells) and aged (*n* = 111 cells and *n* = 108 cells for old_1 and old_2, respectively) samples (**d**). MFI of histone H3 was used as an internal control for normalization. Nuclei were stained with DAPI. Scale bars, 25 μm. **e**, Model for CiC-mediated connection among mitochondrial quality control, chromatin and stemness in aged MSCs. Upon aging, MSCs show chromatin compaction and altered profile of histone marks on promoters and enhancers of genes associated with osteogenesis, resulting in lower osteogenic capacity. Despite global reduction in histone acetylation, aged MSCs contain high levels of acetyl-CoA. However, in the aged cells, acetyl-CoA is trapped inside mitochondria and cannot be used for histone acetylation and lipid biogenesis. This is due to the lower levels of CiC, which, in the aged cells, is incorporated into MDVs and transported to lysosomes for degradation. Therefore, the impaired export of acetyl-CoA from the mitochondria to the cytosol is responsible for the lower osteogenic potential of aged cells. Strikingly, acetate supplementation of aged cells rescues levels of histone acetylation and osteogenic differentiation capacity of aged cells. Distribution of data points in **b** and **d** is shown as violin plots, where the mean is indicated by a solid line and the quartiles are indicated with dashed lines. Statistical significance in **b** and **d** was determined using two-sided unpaired *t*-test.

In the present study we focused on the flux of acetyl-CoA from mitochondria to the nucleus, and we investigated how age-associated changes in the flow of acetyl-CoA affect stem cell function. The data presented here suggest a model (Fig. 6e) whereby aging-driven changes in chromatin structure lead to the transcriptional alterations that are responsible for the decreased osteogenic potential of aged MSCs. We show that, upon aging, there is a shift in the subcellular localization of acetyl-CoA, which affects the epigenetic landscape. In particular, aged MSCs exhibit compartmentalized acetyl-CoA localization in the mitochondria, as a result of enhanced MDV-lysosomal degradation of CiC. Strikingly, restoring histone acetylation, and thus chromatin plasticity, is sufficient to improve the impaired osteogenic capacity of aged MSCs, highlighting the fundamental role of CiC in the regulation of the metabolism–chromatin–osteogenesis axis.

**Extended Data Fig. 7:**
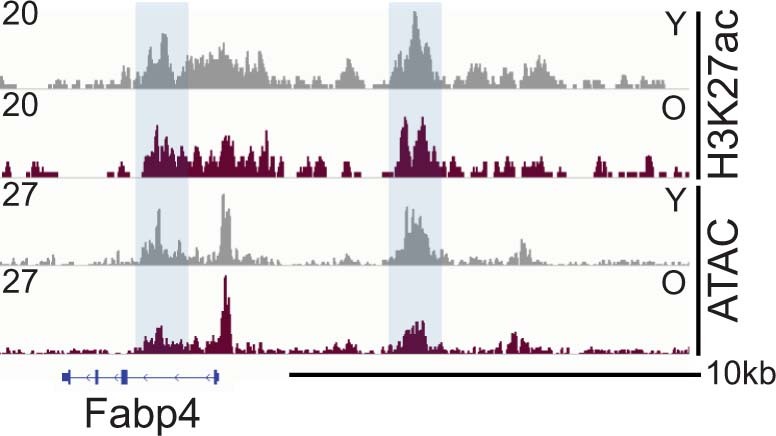
Maintenance of epigenetic marking on adipogenic genes upon aging. IGV Browser screenshot showing H3K27 acetylation read density (combined from n = 3 biologically independent experiments) and ATAC-seq (combined from n = 2 biologically independent experiments) in young and aged MSCs, near *Fabp4* gene.

We show that MSCs from both age groups differentiate efficiently into adipocytes and osteoblasts, whereas aged MSCs show impaired osteogenic capacity (Extended Data Fig. 2c–j). Skewed adipogenic differentiation of aged MSCs at the expense of osteogenesis correlates with previous reports of increased adipocyte content in the aged bone marrow and has been associated with the development of osteoporosis^5,6^. Although our observation about the enhanced adipogenic capacity of purified MSCs is in agreement with a previous report, two recent studies have shown that MSCs exhibit a gene expression profile that is more similar to that of osteocytes^49,50^. However, our data clearly indicate that differentiation to osteoblasts requires a more advanced level of chromatin remodeling. For instance, despite the fact that MSCs isolated from young mice showed active enhancer marking on osteogenesis-involved genes (Fig. 2c–f), extended duration of osteogenesis induction was required for efficient differentiation to osteoblasts, in contrast with the short duration of adipogenesis (Extended Data Fig. 2c–f). One probable explanation for the contradicting phenotypes is the different MSC population used in each case. Opposite to the plastic adherence protocol for purification of a heterogenous MSC population from bone marrow, we isolated and selected a homogenous and highly purified population of MSCs that resides in the bone endosteum^21,51^ and possesses trilineage differentiation potential both in vitro and in vivo^52^. This multilineage differentiation capacity is also supported by our epigenetic data, which showed active enhancer marking not only for genes involved in osteogenesis, but also for those associated with adipogenesis (Extended Data Fig. 7).

Citrate has been suggested recently to be important throughout the osteogenic differentiation of MSCs, via regulating alpha-ketoglutarate production^53–55^. However, we show here that the export of mitochondrial acetyl-CoA to the cytosol in the form of citrate is the critical step for MSC osteogenesis. Remarkably, CiC activity is required before induction of differentiation, as evidenced by the fact that short-term inhibition of CiC function, before stimulation of differentiation, is sufficient to impair osteogenesis (Extended Data Fig. 5g). Our data indicate that this is because the cytosolic/nuclear acetyl-CoA pool is indispensable for the establishment of a plastic chromatin state and the acetylation of enhancers and promoters of osteogenic genes, which allow MSCs to initiate a dedicated differentiation program. These results highlight the central role of the acetyl-CoA-mediated chromatin remodeling in the regulation of stem cell fate decisions. Furthermore, we show that rescuing CiC levels in aged MSCs is sufficient to restore histone acetylation and improves osteogenesis in aged MSCs, demonstrating the importance of CiC in the metabolism-dependent chromatin rearrangements that are required for efficient MSC lineage commitment.

MSCs balance adipogenesis and osteogenesis by integrating environmental signals with their transcriptional regulatory network, and several signaling pathways modulate the chromatin landscape to fine-tune gene expression. Therefore, investigating the crosstalk between epigenetic modifications and other cellular pathways is of high importance, particularly in the context of aging, where many cellular processes undergo profound alterations. In this study, we demonstrate that mitochondrial CiC is degraded in the lysosomes of aged MSCs, which influences stem cell fate decisions via changing the chromatin architecture. Notably, lysosomal CiC degradation in aged MSCs correlates with an increase in the number of MDVs^44^ carrying CiC, and suggests that delivery of CiC to lysosomes is probably mediated via MDVs (Fig. 5). Why is MDV formation enhanced upon aging? So far, MDVs have been studied in cells only after artificial induction of mitochondrial oxidative stress^43,56^. Interestingly, aging is usually accompanied by increased levels of mitochondrial stress^57,58^, which could potentially explain the upregulation of MDV formation in aged MSCs. Nonetheless, given that the MDV area of research is just emerging, future work in this field will shed more light on the specific mechanisms driving CiC selection and incorporation into MDVs.

Finally, the striking observation that short-term treatment of MSCs with acetate resulted in increased histone acetylation and chromatin accessibility, leading to enhanced stem cell differentiation potential, suggests that intervening in the chromatin landscape represents a promising approach to rejuvenate aged MSCs.

## Methods

Ethical approval for all mouse work performed in this study was granted by the Landesamt für Natur, Umwelt und Verbraucherschutz (LANUV) of the federal state of North Rhine-Westphalia, Germany.

Details of all antibodies used in this study are provided in Supplementary Table 1. Models were drawn using BioRender.

### Mouse strains and husbandry

C57BL/6 N mice were bred and cared for in the mouse facility of the Max Planck Institute for Biology of Ageing. Mice were kept at a relative humidity of 50 ± 5%, a room temperature (RT) of 22 ± 2 °C and a light/dark cycle of 12 h (6 a.m. to 6 p.m., with a 15-min twilight period). For our aging experiments and profiling, we used exclusively wild-type male mice at 3–4 months (young cohort) and 18–22 months (old cohort) of age, a range commonly used in age-associated studies.

### Human MSCs

Bone marrow aspirates were obtained from patients undergoing a hemiarthroplasty or total hip arthroplasty at the University Hospital, Limerick, or the Midwestern Orthopaedic Hospital, Croom, Ireland. Ethical approval was obtained from the Health Service Executive and the University of Limerick and all patients gave their informed consent. Osteoporotic female patients were identified following the diagnosis of a femoral neck fragility fracture. Healthy female patients were identified following the diagnosis of a femoral neck fracture where the mechanism of injury was considered sufficient to cause the fracture, that is, trauma. For details on the isolation procedure, see Corrigan et al.^48^

### Endosteal-MSC isolation

To isolate MSCs from their endosteal niche we modified a published protocol^21^ by culturing the bone fragments together with the released cells; this allows the outgrowth of more MSCs from the bone in vitro. Bone chips and cells were cultured under humidified conditions in hypoxia (2% O_2_, 5% CO_2_, 37 °C). On day 12 we performed cell sorting using flow cytometry.

### Cell sorting by flow cytometry

To obtain a purified MSC population, we performed cell sorting by flow cytometry, using the 7AAD dye and the following antibodies: SCA-1-FITC, CD140a-APC, CD45-PE and TER-119-PE. Sorting was performed using BD FACSAria IIu and BD FACSAria IIIu instruments, under low pressure sorting conditions (100 μm nozzle and 20 psi sheath pressure), at 4 °C and the CD45-/TERR-119-/CD140a+/SCA-1+ population was selected. Compensation was done using UltraComp compensation beads (Invitrogen). Sorted MSCs were cultured under humidified conditions in hypoxia (2% O_2_, 5% CO_2_, 37 °C) and maintained in culture for a maximum of four to five passages (~4 weeks after isolation).

To characterize MSCs from young and old mice with respect to their marker expression profile, cells were stained as described above with the following antibodies: SCA-1-FITC, CD140a-APC, CD44-PE, CD29-PE, CD45-PE, CD34-FITC, CD31-APC and TERR-119-PE, and analyzed using the BD FACSCanto II instrument. This experiment was done with purified MSCs at passage two, and with purified MSCs after 4 weeks of cell culture (Extended Data Fig. 1).

### Plasmid construction, generation of lentivirus and MSCs transduction

Construct pLVX-Puro-slc25a1 was generated by cloning the Slc25a1 cDNA sequence fused to C-terminal FLAG epitope (synthesized by Geneart) into a pLVX-Puro vector (Takarabio) between *Xho*I and *Xba*I restriction sites. Lentivirus was generated by cotransfection of 293 T cells (cultured in DMEM-GlutaMAX, 10% FBS, 1× penicillin/streptomycin (pen-strep)) with pLVX-Puro vector or pLVX-Puro-Slc25a1-FLAG, pMD2.G and psPAX2 vectors (pMD2.G and psPAX2 were a gift from D. Trono; Addgene plasmid catalog nos. 12259 and 12260, respectively). Specifically, 2.8 million HEK cells were seeded in a 10 cm plate. The next day, a transfection mix of 500 ml 2×HBS, 62 ml CaCl_2_ 2 M, 10 mg pLVX, 5.2 mg pMD2.G and 5.2 mg psPAX2 was incubated for 30 min at RT and subsequently added to the cells. After 16 h, the medium was changed for 6 ml fresh DMEM-GlutaMAX, 10% FBS, 1× pen-strep. After 72 h, the supernatant was collected, spun at 500 g for 5 min and filtered through a 0.45 µM filter.

A 1:1 virus:medium ratio along with 4 µg ml^−1^ Polybrene was used to transduce MSCs. Medium was changed after 18 h. Puromycin (2 µg ml^−1^) was added after 72 h to positively select transduced cells.

### qRT-PCR

Cells were lysed with QIAzol (QIAGEN) and total RNA was extracted using RNA extraction kit (Direct-zol RNA MiniPrep; Zymoresearch) following the manufacturer’s protocol. This was followed by cDNA synthesis using Maxima H Minus cDNA synthesis master mix (Thermo Scientific), according to the manufacturer’s instructions. Subsequent qRT-PCR was performed using SYBR-Green chemistry (Roche) on a Light Cycler 96 instrument (Roche). Data were analyzed and further processed in Microsoft Excel and Prism8. Fold change in gene expression over control samples was calculated using the ΔΔCq method, where β-actin Cq values were used as internal control. All reactions were run in three technical replicates and averaged. Experiments were performed three times independently and merged results are shown. Sequences of the qPCR primers for *CiC*, *ACAN* and *β-actin* genes are provided in Supplementary Table 2.

### Measurement of mtDNA content

RT-qPCR of mtDNA-encoded genes was used to estimate the mtDNA content. Cells were lysed with DNA lysis buffer and lysates were incubated with Proteinase K (Sigma-Aldrich). Phenol (Roth) was then added to the samples to denature proteins and an equal amount of chloroform (Roth) was then added to remove residual phenol. To precipitate DNA, the aqueous phase was collected again, samples were incubated overnight at −80 °C with sodium acetate (pH 5.2; Invitrogen) and 100% ethanol (Roth) and the precipitated DNA was resuspended in nuclease-free water. RT-qPCR was performed as described above. All reactions were run in three technical replicates and averaged. Experiments were performed three times independently and merged results are shown. Sequences of the qPCR primers for *cox-1* and *atp6* genes are provided in Supplementary Table 2.

### 5-EU labeling

The 5-EU label was added to the cells, diluted in complete culture medium from a 100 mM stock in DMSO. After 7 h of labeling with 1 mM 5-EU, cells were fixed in fixation buffer (125 mM PIPES pH 6.8, 1 mM MgCl_2_,10 mM EGTA, 0.2% TritonX-100 and 3.7% formaldehyde), for 30 min at RT. For 5-EU detection, fixed cells were stained for 30 min at RT with staining buffer (100 mM Tris pH 8.5, 1 mM CuSO_4_, 38.4 µM fluorescent azide 488 and 100 mM ascorbic acid, added last from a 0.5 M stock). The samples were then rinsed several times with 0.5% TBS-TritonX-100 and then stained with 4,6-diamidino-2-phenylindole (DAPI); 30–40 stained cells per sample were imaged using the Confocal SP8X microscope with a ×63 objective.

### Mitotracker staining

Cells were resuspended in prewarmed (37 °C) staining solution containing the MitoTracker Deep Red FM probe (Thermo Fischer Scientific) at a final dilution of 1:15,000 (in a-MEM medium without FBS and phenol red). Cells were incubated with the staining solution for 30 min at 37 °C. After addition of DAPI (Invitrogen) for dead-cell exclusion right before measurement, cells were analyzed by flow cytometry (BD FACSCANTO II cytometer; BD Biosciences). Data were collected using FACS-Diva software and analyzed using FlowJo software.

### Measurement of bone parameters by μCT

Mouse femurs were scanned with a high resolution µCT scanner (SkyScan 1176, Bruker) with an isotropic voxel size of 8.8 µm^3^. The X-ray settings for each scan were 50 kV and 200 µA using a 0.5 mm aluminum filter. All scans were performed over 360° with a rotation step of 0.3° and a frame averaging of 1. Images were reconstructed and analyzed using NRecon and CTAn software, respectively (Bruker). Trabecular and cortical bone regions of distal femurs were selected with reference to the growth plate (0.44–2.2 and 2.2–2.64 mm from growth plate, respectively). Bone mineral density was determined on the basis of calibration with two phantoms of known density (Bruker), which were scanned under the same conditions as the bone samples.

### Oxygen consumption rate

The SeaHorse XF96 extracellular Flux Analyzer (Agilent Technologies) was used to determine oxygen consumption rates (OCR) in young and aged cells. A total of 20,000 cells were seeded in 96-well SeaHorse plates after coating them with 10% gelatin−90% poly-l-lysine solution. To measure endogenous fatty acid oxidation, on the day of the experiment cells were treated either with DMSO or with 100 μM etomoxir (Sigma-Aldrich) and incubated for 1 h at 37 °C, in a humidified 5% CO_2_, 2% O_2_ incubator. They were then washed twice with the assay medium (XF-DMEM medium supplemented with 10 mM glucose, 1 mM pyruvate and 2mM l-glutamine) and incubated for 1 h before loading into the XF Analyzer, in a non-CO_2_-containing incubator.

To assess exogenous fatty acid oxidation, cells were incubated overnight with substrate-limited medium (DMEM without glucose and phenol red, supplemented with 0.5 mM glucose, 0.5 mM l-carnitine, 1 mM Glutamax, 1% FBS). On the day of the experiment, cells were incubated with the assay medium (substrate-limited medium supplemented with 2 mM glucose, 0.5 mM l-carnitine) for 1 h in a non-CO_2_-containing incubator. BSA-palmitate was added to the wells before running the assay.

Following measurements of resting respiration, cells were injected subsequently with 20 μM oligomycin (or 1.5 μM for the palmitate experiment), 5 μM FCCP (or 2 μM for the palmitate experiment) and 5 μM rotenone/antimycin (or 0.5 μM for the palmitate experiment); all drugs were from Agilent Technologies. Each measurement was taken over a 2-min interval followed by 2 min of mixing and 2 min of incubation. Three measurements were taken for the resting OCR: after oligomycin treatment, after FCCP and after rotenone/antimycin A treatment. Values were normalized to protein concentration using a Bradford kit and were plotted using the Wave 2.4 and the Prism8 software programs.

### Assessment of proliferation rate

To compare the proliferation rate of young and aged MSCs, we used the CyQUANT NF Cell Proliferation Assay Kit (Invitrogen), according to the manufacturer’s instructions.

### Colony-forming unit assay

To determine the efficiency with which MSCs form colonies, 100 cells were seeded in a six-well plate. After 10–12 days of cell culture, cells were stained with 1% (v/v) Crystal Violet solution (Sigma-Aldrich) and visible colonies were counted. Images were acquired on a CanonScan 9000 F Mark II scanner.

### Differentiation assays

#### Differentiation to adipocytes

Adipogenic differentiation was induced by culturing cells in differentiation medium (a-MEM medium supplemented with (1) 1 μM dexamethasone (Sigma-Aldrich) (2) 1 μM IBMX (Sigma-Aldrich), (3) 10 μg ml^−1^ insulin (Sigma-Aldrich) and (4) 100 μM indomethacin (Sigma-Aldrich)), for 8–10 days. For BTA treatment of young cells, 1 mM BTA (Sigma-Aldrich) was added to the control medium 3 days before differentiation. Adipocytes were detected by Oil red-O staining (Sigma-Aldrich) and images were acquired with bright-field microscope (EVOS FL AUTO), using the ×20 objective. Oil red O staining was quantified by extracting the color in isopropanol and measuring the absorbance at 500 nm.

#### Differentiation to osteoblasts

Osteogenesis was induced by culturing cells in differentiation medium (a-MEM medium supplemented with (1) 100 nM dexamethasone, (2) 10 mM beta-glycerophosphate (Sigma-Aldrich) and (3) 100 μM ascorbic acid (Sigma-Aldrich)), for 8–12 days. For BTA treatment of young cells, 1 mM BTA was added to control medium for 3 days before the induction of differentiation. For acetate treatment of aged cells, 5 mM of sodium acetate was added in the medium 3 days before the induction of differentiation. Osteoblasts were detected by Alizarin red S (Sigma-Aldrich) staining and images were acquired with bright-field microscope (EVOS FL AUTO), using the ×20 objective. Quantification of the Alizarin red S staining was done in ImageJ, after imaging three to five randomly selected fields of each replicate.

#### Differentiation to chondrocytes

Chrondrogenesis was induced by culturing cells in differentiation medium (DMEM medium supplemented with (1) 100 nM dexamethasone, (2) 1% ITS (Sigma-Aldrich), (3) 10 μM ascorbic acid, (4) 1 mM sodium pyruvate (Gibco), (5) 50 μg ml^−1^ proline (Sigma-Aldrich) and (6) 20 nm ml^−1^ TGFb3 (Sigma-Aldrich)) media for 12–14 days.

Confirmation of chondrogenesis was performed by extracting RNA and running qPCR analysis for chondrogenic markers, as described above.

### Immunofluorescence experiments

Cells were fixed for 15 min at 37 °C with 3.7% v/v formaldehyde in a-MEM medium, permeabilized with 0.1% TritonX-100 (Roth) in PBS for 10–15 min and blocked with 5% BSA-PBS (Roth) for 50 min. For histone acetylation immunostaining, cells were incubated for 10 min with CSK buffer (10 mM PIPES pH 6.8, 100 mM NaCl, 300 mM sucrose, 3 mM MgCl_2_, 1 mM EDTA, 0.5% Triton-X100), on ice. They were then fixed for 20 min at RT with fixation buffer (1× PBS, 4% paraformaldehyde, 2% sucrose, 0.4 mM NaOH in H_2_O) and blocked with 3% BSA-PBS, for 1 h at RT. Samples were then incubated, overnight at 4 °C with the indicated primary antibodies diluted in 5% BSA-PBS. Following this, they were incubated for 45 min with the appropriate secondary fluorescent antibodies diluted in 5% BSA-PBS, protected from light. Cells were mounted using Roti-Mount FluorCare mounting medium (HP20.1, Roth) containing DAPI. Images were acquired using ×40 and ×63 objective lenses on an SP8X Leica confocal microscope.

For triple immunostaining of MDVs, cells were fixed in fixation buffer (6% paraformaldehyde in PBS) for 20 min at 37 °C. They were then permeabilized with 0.1% Triton-X100 in PBS for 10 min at RT and blocked with 10% FBS-PBS for 45 min at RT. Following blocking, cells were incubated with primary antibodies diluted in 5% FBS-PBS, overnight at 4 °C and with the respective fluorophore-conjugated secondary antibodies diluted in 5% FBS-PBS for 1 h at RT, protected from light. Cells were mounted as described above. Images were acquired using a ×100 objective lens on an SP8-X Leica confocal microscope.

### Electron microscopy

Samples were fixed in fixation buffer (2% glutaraldehyde, 2.5% sucrose, 3 mM CaCl_2_, 100 mM HEPES pH 7.4), for 30 min at RT and 30 min at 4 °C, washed with 0.1 M sodium cacodylate buffer (1% osmium, 1.25% sucrose and 10 mg ml^−1^ potassium ferrocyanid in 0.1 M sodium cacodylate), incubated for 1 h on ice OSO4, and washed again with 0.1 M sodium cacodylate buffer. After ethanol washes, samples were incubated with EPON and embedded. Sections (70 nm) were cut using Leica Ultracut and put on negatively stained grids with 1.5 % uranylacetate in water at 37 °C in the dark. Images were acquired using a JEM 2100 plus microscope (JEOL).

### Western blot experiments

Cells were lysed with RIPA buffer supplemented with 5 mM sodium butyrate and 1× Protease Inhibitor Cocktail (Thermo Scientific). Protein extracts were resolved using SDS-PAGE and transferred to nitrocellulose membranes, using the Trans-Blot Turbo blotting apparatus and reagents (all from Bio-Rad). Protein transfer was confirmed by Ponceau S staining (Sigma-Aldrich). The membranes were then blocked using 5% nonfat dry milk in Tris-buffered saline, 0.1% Tween20 (TBS-T) for 1 h at RT and were probed with the indicated primary antibodies, diluted in 3% milk in TBS-T at 4 °C overnight. Membranes were then incubated with horseradish peroxidase-conjugated secondary antibodies diluted in 5% BSA in TBS-T, for 1 h at RT. Detection was performed providing fresh horseradish peroxidase-substrate solution (Luminol Enhancer Solution/Peroxide Solution; Promega) and exposing of membranes for specific time periods to photographic film using a Curix60 instrument (Agfa).

### Measurement of glucose, lactate and pH

Measurement of glucose, lactate and pH in MSC media was performed using a Vi-CELL MetaFLEX instrument (Beckman Coulter) according to manufacturer’s instructions. All measurements were done in triplicates and averaged.

### Targeted LC-MS analysis of acetyl-CoA

Metabolite extraction from each sample was performed using a mixture of 40:40:20 (v:v:v) of prechilled (−20 °C) acetonitrile:methanol:water (OptimaTM LC/MS grade, Thermo Fisher Scientific) and protein concentration was used for normalization (BCA Protein Assay Kit, Thermo Fisher Scientific). For analysis of acetyl-CoA, the extracted metabolites were resuspended in UPLC-grade acetonitrile:water (80:20 (v:v), OptimaTM LC-MS-grade, Thermo Fisher Scientific). The samples were analyzed on an Acquity iClass UPLC (Waters), using a SeQuant ZIC-HILIC 5 µm polymer 100 ×2.1 mm column (Merck) connected to a Xevo TQ-S (Waters) triple quadrupole mass spectrometer. An 8 µl sample of the resuspended metabolite extract was injected onto the column and separated using a flow rate of 500 µl min^−1^ of buffer A (10 mM ammonium acetate, 0.1% acetic acid) and buffer B (acetonitrile) using the following gradient: 0–0.5 min 20% A; 0.5–1.4 min 20–35% A; 1.4–2.5 min 35–65% A. After 2.5 min, the system is set back to 20% A and re-equilibrated for 2.5 min.

The eluted metabolites were detected in positive ion mode using ESI MRM (multireaction monitoring) applying the following settings: capillary voltage 1.5 kV, desolvation temperature 550 °C, desolvation gas flow rate 800 L h^−1^, collision cell gas flow 0.15 ml min^−1^. The following MRM transitions were used for relative compound quantification of acetyl-CoA m/z precursor mass (M+H+) 810, fragment mass (M+H+) m/z 303 using a cone voltage of 98 V and a collision energy of 28 V. For each compound, two further fragments were monitored as qualitative controls for compound identity. Data analysis and peak integration were performed using the TargetLynx Software (Waters).

### RNA-seq

Total RNA was isolated using the RNA extraction kit (Direct-zol RNA MiniPrep, Zymoresearch), following the manufacturer’s protocol. Once RNA quality and integrity were verified, RNA libraries were created using the NEBNext Ultra II Directional RNA Library Prep Kit for Illumina at the MPI for Plant Breeding. Libraries were sequenced as single-end 150 bp reads on Illumina HiSeq 4000. The sequenced reads of RNA-seq dataset were processed using zUMIs (v.2.2.1)^59^ with STAR (v.2.6.1a)^60^, Samtools (v.1.9)^61^ and featureCounts from Rsubread (v.1.32.4)^62^. The reads were mapped to the mouse genome (mm10) with the ensembl annotation v.GRCm38.91. The generated count matrix was further analyzed using R (v.3.5.1). First, genes were filtered using the ‘filterByExpr’ function of edgeR^63^ with the min.count =5. The differential gene expression analysis between young and old mice was carried out using the limma-trend^64^ approach at the adjusted *P*-value of 0.05. Obtained sets of genes were further analyzed, for example through GO enrichment analysis.

### ATAC-seq

We performed ATAC-seq on 50,000 cells per sample, as described previously^4^. DNA concentration of the libraries was measured using Qubit, and library quality was assessed by running samples on the TapeStation.

The fastq files of sequenced reads were mapped to the mouse genome (mm10) using local alignment using bowtie2 (ref. ^65^) with parameters -x mm10 and -X 2000. The resulting BAM files were sorted, indexed using Samtools (v.1.3.1) and duplicates were removed using MarkDuplicates of Picard Tools. The peaks were called using chromstaR^66^ R package in differential mode between young and old with the binsize of 300, stepsize of 100 and 15 as minimum mapping quality threshold. The per sample peak RPKM (reads per kilobase of transcript per million mapped reads) table was pulled out from the chromstaR model and we performed differential accessibility analysis between young and old using edgeR^63^. The normalizing factors were calculated using ‘RLE’ method in ‘calcNormFactors’, tagwise dispersion trend was estimated using the default parameters in ‘estimateDisp’ function and a generalized linear model was then fit on the data using ‘glmQLFit’ function in robust mode.

The peaks called by chromstaR were then used in nucleoATAC^25^ for nucleosome positioning. For visualization purpose, the replicates were merged using ‘samtools merge’ and the bigwig files were generated using ‘bamCoverage–normalizeUsing RPGC’.

### CUT&RUN

We performed CUT&RUN on 100,000 cells per sample adapting a previously described protocol^8^. DNA was purified using a Zymo DNA clean concentrator kit and DNA was eluted in the elution buffer provided with the kit.

For library construction, we modified a previously described protocol^51^. DNA concentration of the libraries was measured using Qubit, and library quality was assessed by running samples on the TapeStation.

The fastq reads were mapped to mm10 genome using bowtie2 (ref. ^65^) and duplicates were then removed using MarkDuplicates program of Picard Tools. For mapping spike-in fragments to yeast, the ‘–no-overlap–no-dovetail’ options were set and mapped to a repeat-masked version of the yeast genome (R64) to avoid crossmapping of the mouse genome to that of the yeast genome. The peaks were then called using the chromstaR^66^ package in differential mode between young and old for each histone mark (H3K27ac, H3K27me3 and H3ac) with the binsize of 1,000, stepsize of 500 and 15 as minimum mapping quality threshold. We performed differential analysis between young and old for each mark using edgeR^65^ as described under the ATAC-seq analysis section. For visualization purposes, the replicates were merged using ‘samtools merge’ and the bigwig files were generated using ‘bamCoverage–normalizeUsing RPGC’. The differential regions in the H3AC histone mark data were identified using chromstaR in differential mode with eight states. The segments with the different states in old, acetate-treated old and young samples were obtained from multivariate peak calls.

### DAStk analysis

The open chromatin regions were called from the resulting BAM files from mononucleosome, dinucleosome, trinucleosome and nucleosome-free regions using MACS2, with settings–shift −100–extsize 200 -B–nomodel–format BAMPE. Any ATAC-seq peaks overlapping blacklisted regions for the mm10 mouse reference genome (ENCODE ENCFF547MET) were excluded from the analysis.

We used mouse motifs from the HOCOMOCO^67^ database v.11, and scanned for coordinates of motif sites in the mm10 mouse reference genome using FIMO^68^ at a p-value threshold of 10-6. DAStk (https://github.com/Dowell-Lab/DAStk) was used to predict changes in transcription factor activity, using a *P*-value <0.05 cutoff for statistical significance.

### Image acquisition and processing

Bright-field images were acquired using the Evos FL Auto 2 microscope. Quantification of the AlizarinrRed S staining was done in ImageJ software; measurement of intensity was done after making RGB stacks for each image and applying the same thresholds to all samples from each experiment. Immunofluorescent images were acquired using confocal SP8-X and SP8-DLS microscopes (Leica). All immunofluorescent images were processed identically in ImageJ; in particular, images are shown after background subtraction (rolling ball radius:50) and noise despeckle.

### Quantification and statistical analysis

Except for epigenome analysis, all other graphs were generated in GraphPad Prism8. For all bar graphs, results are shown as mean ± s.e.m. For quantification of images where more than ten cells were taken into account, distribution of data points is shown as violin plots, where the mean is indicated by a solid line and quartiles are indicated with dotted lines. *P*-values for box plots of sequencing data in (Fig. 1c) and (Fig. 4d) were determined using two-sided Wilcoxon test. Statistical significance was determined using two-sided unpaired *t*-test, unless otherwise specified, and the exact *P*-values are indicated on each plot. We performed GO enrichment analysis (Fig. 1f, Fig. 2e, j, Fig. 4d and Extended Data Fig. 2g) using Metascape^69^, which calculates *P*-values on the basis of accumulative hypergeometric distribution.

### Reporting Summary

Further information on research design is available in the Nature Research Reporting Summary linked to this article.

## Supplementary information

Supplementary Tables 1 and 2

Reporting Summary

## Data Availability

ATAC-seq, RNA-seq and CUT&RUN-seq data are available at GEO, accession code: GSE 143580. Source data are provided with this paper.

## Source data

Source Data Fig. 1. Statistical source data for Fig. 1.
Source Data Fig. 2. Statistical source data for Fig. 2.
Source Data Fig. 3. Statistical source data for Fig. 3.
Source Data Fig. 3. Unprocessed western blots for Fig. 3.
Source Data Fig. 4. Statistical source data for Fig. 4.
Source Data Fig. 5. Statistical source data for Fig. 5.
Source Data Fig. 6. Statistical source data for Fig. 6.
Source Data Extended Data Fig. 1. Statistical source data for Extended Fig. 1.
Source Data Extended Data Fig. 2. Statistical source data for Extended Fig. 2.
Source Data Extended Data Fig. 3. Statistical source data for Extended Fig. 3.
Source Data Extended Data Fig. 4. Statistical source data for Extended Fig. 4.
Source Data Extended Data Fig. 4. Unprocessed western blots for Extended Fig. 4.
Source Data Extended Data Fig. 5. Statistical source data for Extended Fig. 5.
Source Data Extended Data Fig. 6. Statistical source data for Extended Fig. 6.
Source Data Extended Data Fig. 6. Unprocessed western blots for Extended Fig. 6.
