# Supplementary material for: Chromatin remodeling due to degradation of citrate carrier impairs osteogenesis of aged mesenchymal stem cells
Source: Nat Aging. 2021 Sep 13;1(9):810–25. doi: 10.1038/s43587-021-00105-8 (PMC10154229; doi:10.1038/s43587-021-00105-8)
Supplement: Supplementary file 1 — Supplementary Tables 1 and 2 [file 43587_2021_105_MOESM1_ESM.pdf]

nature portfolio

https://doi.org/10.1038/s43587-021-00105-8

Supplementary information

Chromatin remodeling due to degradation of citrate carrier impairs osteogenesis of aged mesenchymal stem cells

In the format provided by the authors and unedited

Supplementary Table 1

Antibody	Supplier	Catalogue Numbe	LOT	Dilution
Rat monoclonal anti-CD140a-APC	eBioscience	17140181	1996382	1/1000
Rat monoclonal anti-Sca-1-FITC	eBioscience	11598182	1923823	1/100
Rat monoclonal anti-Terr-119-PE	eBioscience	12592182	4313528	1/100
Mouse monoclonal anti-CD45-PE	Life Technologies	A16325	2051673	1/100
Rat monoclonal anti-CD34-FITC	eBioscience	11034181	E00263-1631	1/100
Goat polyclonal anti-CD31-APC	eBioscience	17031180	E071241632	1/100
Rat monoclonal anti-CD140a-APC	invitrogen	17140181	2213095	1/100
Armenian hamster monoclonal anti-CD29-PE	eBioscience	120291-81	E011811636	1/100
Rat monoclonal anti-CD44-PE	eBioscience	12044181	E012381632	1/100
Rabbit polyclonal anti-pan-acetylated-Lysine	Cell Signaling Technology	9441S	14	1/400
Rabbit polyclonal anti-Fatty Acid Synthase	Cell Signaling Technology	3189S	2	1/1000
Rabbit polyclonal anti-CBP	Cell Signaling Technology	7389S	3	1/1000
Rabbit monoclonal anti-AceCS1	Cell Signaling Technology	3658T	2	1/1000
Mouse monoclonal anti-Histone H3	Cell Signaling Technology	14269	6	1/400
Rabbit polyclonal anti-Histone H3 K27ac	Active Motif	39133	31416013	1/300
Rabbit polyclonal anti-Histone H3 K27me3	Active Motif	39155	31814017	1/300
Rabbit polyclonal anti-ACC1	ProteinTech	21923-1-AP	48856	1/1000
Rabbit polyclonal anti-ACLY	ProteinTech	15421-1-AP	6573	1/1000
Mouse monoclonal anti-TOMM20	Santa Cruz Biotechnology	sc-17764	k0117	1/100
Mouse monoclonal anti-TOMM20	Sigma-Aldrich	WH0009804M1	L4221-4F3	1/200
Mouse monoclonal anti-b-actin	Santa Cruz Biotechnology	sc-47778	A2418	1/1000
Mouse monoclonal anti-PDH1	Abcam	110333	GR3286545-4	1/200
Rabbit polyclonal anti-LONP1	Sigma-Aldrich	HPA002192	D118477	1/1000
Rabbit polyclonal anti-GCN5	ProteinTech	14983-1-AP		1/1000
Rat monoclonal anti-LAMP2	DSHB	ABL-93	LOT3443366	1/200
Rabbit polyclonal anti-PGAM5	Sigma Prestige	HPA036978	G119179	1/1000
Rabbit polyclonal anti-PARL	Sigma-Aldrich	AV44851-50UG	QC14615	1/1000
Rabbit polyclonal anti-pan-acetylated Histone H3	Active Motif	39139	34315006	1/100
Rabbit polyclonal anti-pan-acetylated Histone H4	EMD Millipore	06-866	3045752	1/100
Mouse monoclonal anti-Histone H4	abcam	31830	GR3204774-3	1/200
Rabbit polyclonal anti-Citrate Carrier	abcam	99168	GR205394-12	1/300
Rabbit polyclonal anti- Citrate Carrier	ProteinTech	15235-I-AP	00006349	1/100
Rabbit monoclonal anti-FLAG	Cell signalling Technology	14793S	5	1/500
Donkey polyclonal anti-alexa Fluor 488	invitrogen	A21206	2156521	1/500

Rabbit polyclonal anti-alexa Fluor 594	life technologies	A21207	1602780	1/500
Goat polyclonal anti-alexa Fluor 488	invitrogen	A21121	1889303	1/500
Goat polyclonal anti-alexa Fluor 647	invitrogen	A21241	2154867	1/500
Mouse monoclonal anti-OPA1	BD Biosciences	612606	4164691	1/1000
Rabbit polyclonal anti-YME1L	ProteinTech	11510-1-AP	RRID:AB_2217	1/1000
Rabbit Polyclonal anti-STARD7	ProteinTech	15689-1-AP	RRID:AB_2197	1/1000

**Supplementary Table 2**
**qPCR primer sequences**
Slc25a1 (encoding citrate carrier)
5’ GGAGAGGACTATTGTGCGGTCT 3’
5’ CCCGTGGAAAAATCCTCGGTAC 3’
Acan
5’ CGTTGCAGACCAGGAGCAAT 3’
5’ CGGTCATGAAAGTGGCGGTA 3’
β-actin
5’ CTGCGCTGGTCGTCG 3’
5’ CACGATGGAGGGGAATACAG 3’
atp6
5’ GGCACCTTCACCAAAATCAC 3’
5’ CGGTTGTTGATTAGGCGTTT 3’
cox-1
5’ AGGTTGGTTCCTCGAATGTG3’
5’ GCCTTTCAGGAATACCACGA 3’
