# Supplementary material for: Chromatin remodeling due to degradation of citrate carrier impairs osteogenesis of aged mesenchymal stem cells
Source: Nat Aging. 2021 Sep 13;1(9):810–25. doi: 10.1038/s43587-021-00105-8 (PMC10154229; doi:10.1038/s43587-021-00105-8)
Supplement: Supplementary file 2 — Reporting Summary [file 43587_2021_105_MOESM2_ESM.pdf]

nature research

Corresponding author(s): Peter Tessarz

Last updated by author(s): 2021/07/07

Reporting Summary

Nature Research wishes to improve the reproducibility of the work that we publish. This form provides structure for consistency and transparency in reporting. For further information on Nature Research policies, see Authors & Referees and the Editorial Policy Checklist.

Statistics

For all statistical analyses, confirm that the following items are present in the figure legend, table legend, main text, or Methods section.

n/a	Confirmed	
☐	☒	The exact sample size (*n*) for each experimental group/condition, given as a discrete number and unit of measurement
☐	☒	A statement on whether measurements were taken from distinct samples or whether the same sample was measured repeatedly
☐	☒	The statistical test(s) used AND whether they are one- or two-sided<br>*Only common tests should be described solely by name; describe more complex techniques in the Methods section.*
☒	☐	A description of all covariates tested
☒	☐	A description of any assumptions or corrections, such as tests of normality and adjustment for multiple comparisons
☐	☒	A full description of the statistical parameters including central tendency (e.g. means) or other basic estimates (e.g. regression coefficient) AND variation (e.g. standard deviation) or associated estimates of uncertainty (e.g. confidence intervals)
☐	☒	For null hypothesis testing, the test statistic (e.g. *F*, *t*, *r*) with confidence intervals, effect sizes, degrees of freedom and *P* value noted<br>*Give P values as exact values whenever suitable.*
☒	☐	For Bayesian analysis, information on the choice of priors and Markov chain Monte Carlo settings
☒	☐	For hierarchical and complex designs, identification of the appropriate level for tests and full reporting of outcomes
☒	☐	Estimates of effect sizes (e.g. Cohen's *d*, Pearson's *r*), indicating how they were calculated

Our web collection on statistics for biologists contains articles on many of the points above.

Software and code

Policy information about availability of computer code

Data collection	zUMIs (version 2.2.1); STAR (version 2.6.1a), samtools (version 1.9); featureCounts from Rsubread (version 1.32.4); R (version 3.5.1); edgeR; bowtie2; chromstaR
Data analysis	Prism GraphPad 8 Software (version: 8.4.2) was used to generate graphs and perform statistical analysis. FlowJo Software (version: 10.7.2) was used for flow cytometry data analysis. ImageJ Software (version: 2.0.0-rc-43/1.50e) was used for imaging data analysis. Wave controller Software from Agilent Technologies (version 2.2.0) was used for SeaHorse data analysis. Integrative Genomic Viewer Software (IgV version 2.7.2) was used for gene track views. BD FACSDiva Software (version 8.0.1) was used for cell sorting analysis.<br>Standard and published pipelines (see Data collection) and software were used for NGS data analysis. Packages were cited accordingly.

For manuscripts utilizing custom algorithms or software that are central to the research but not yet described in published literature, software must be made available to editors/reviewers. We strongly encourage code deposition in a community repository (e.g. GitHub). See the Nature Research guidelines for submitting code & software for further information.

Data

Policy information about availability of data

All manuscripts must include a data availability statement. This statement should provide the following information, where applicable:

- Accession codes, unique identifiers, or web links for publicly available datasets
- A list of figures that have associated raw data
- A description of any restrictions on data availability

NGS (RNA- and ATAC-seq as well as CUT&RUN-seq) data that support the findings of this study are available at gene expression omnibus, GEO143580; link: https://www.ncbi.nlm.nih.gov/geo/query/acc.cgi?acc=GSE143580

Field-specific reporting

Please select the one below that is the best fit for your research. If you are not sure, read the appropriate sections before making your selection.

☒ Life sciences ☐ Behavioural & social sciences ☐ Ecological, evolutionary & environmental sciences

For a reference copy of the document with all sections, see nature.com/documents/nr-reporting-summary-flat.pdf

Life sciences study design

All studies must disclose on these points even when the disclosure is negative.

Sample size	Sample size was determined based on previously published reports (Le et al., Nat Cell Biol., 2016; Wu et al., Nature 2016; Mylonas and Tessarz, Life Sci. Alliance, 2018; Dawson et al., Cell Rep., 2012). No statistical method was used to predetermine sample size.
Data exclusions	The only pre-established exclusion criterion was for replicates that were found to be technically flawed or determined by statistical tests to contain a legitimate outlier data point. When any of the above occurred, the entire replicate was omitted and the whole experiment was repeated, when feasible. The number of final replicates used for each analysis is extensively described in the next section.
Replication	Taking into consideration the above exclusion criterion, all experiments were successfully reproduced at least twice. Final number of biological replicates is clearly indicated in each figure legend.
Randomization	Experimental groups were based on age and/or treatment
Blinding	None of our experiments were blinded, since results were inherently objective and unbiased. Results were subjected to binary interpretation, based on statistical tests. Furthermore, analyses could not be blinded because experiments were performed and analyzed by the same researchers.

Reporting for specific materials, systems and methods

We require information from authors about some types of materials, experimental systems and methods used in many studies. Here, indicate whether each material, system or method listed is relevant to your study. If you are not sure if a list item applies to your research, read the appropriate section before selecting a response.

Materials & experimental systems

n/a	Involved in the study
☐	☒ Antibodies
☐	☒ Eukaryotic cell lines
☒	☐ Palaeontology
☐	☒ Animals and other organisms
☐	☒ Human research participants
☒	☐ Clinical data

Methods

n/a	Involved in the study
☐	☒ ChIP-seq
☐	☒ Flow cytometry
☒	☐ MRI-based neuroimaging

Antibodies

Antibodies used

CD140a-APC (eBioscience, 17140181; LOT 1996382; 1:1000)
Sca-1-FITC (eBioscience; 11598182; LOT 1923823; 1:100)
Terr-119-PE (eBioscience; 12592182; LOT 4313528; 1:100)
CD45-PE (Life Technologies; A16325; LOT 2051673; 1:100)
CD34-FITC(eBioscience; 11034181; LOT E00263-1631; 1:100)
CD31-APC(eBioscience; 17031180; LOT E071241632; 1:100)
CD140a-APC(invitrogen; 17140181; LOT 2213095; 1:100)
CD29-PE(eBioscience; 120291; LOT E011811636; 1:100)
CD44-PE(eBioscience; 12044181; LOT E012381632; 1:100)
pan-acetylated-Lysine (Cell Signaling Technology; 9441S; LOT 14 ; 1:400)
Fatty Acid Synthase (Cell Signaling Technology; 3189S; LOT 2; 1:1000)
CBP (Cell Signaling Technology; 7389S; LOT 3; 1:1000)
AceCS1 (Cell Signaling Technology; 3658T; LOT 2; 1:1000)
Histone H3 (Cell Signaling Technology; 14269; LOT 6; 1:400)
Histone H3 K27ac (Active Motif, 39133; LOT 31416013; 1:300)
Histone H3 K27me3 (Active Motif, 39155; LOT 31814017; 1:300)
ACC1 (ProteinTech; 21923-1-AP; LOT 00048856; 1:1000)
ACLY (ProteinTech; 15421-1-AP; LOT 00006573; 1:1000)
TOMM20 (Santa Cruz Biotechnology; sc-17764; LOT k0117; 1:100)
TOMM20 (Sigma-Aldrich; WH0009804M1; LOT L4221-4F3; 1:200)

b-actin (Santa Cruz Biotechnology; sc-47778; LOT A2418; 1:1000)
PDH1 (Abcam, 110333, LOT GR3286545-4; 1:200)
LONP1 (Sigma, HPA002192; LOT D118477; 1:1000)
GCN5(ProteinTech; 14983-1-AP; 1:1000)
LAMP2(EMD Millipore; ABL-93; LOT 3443366; 1:200)
PARL (Sigma, AV44851-50UG; LOT QC14615; 1:1000)
pan-acetylated Histone H3 (Active Motif; 39139; LOT 34315006; 1:100)
pan-acetylated Histone H4 (EMD Millipore; 06-866; LOT 3045752; 1:100)
Histone H4 (abcam; 31830; LOT GR3204774-3; 1:200)
Citrate Carrier (abcam; 99168; LOT GR205394-12; 1:300 and ProteinTech; 15235-I-AP; LOT 00006349; 1:100)
a-FLAG (Cell signalling Technology; 14793S; LOT 5; 1:500)
Donkey polyclonal anti-alexa Fluor 488 (invitrogen; A21206; LOT 2156521; 1:500)
Rabbit polyclonal anti-alexa Fluor 594 (life technologies; A21207; LOT 1602780; 1:500)
Goat polyclonal anti-alexa Fluor 488 (invitrogen; A21121; LOT 1889303; 1:500)
Goat polyclonal anti-alexa Fluor 647 (invitrogen; A21241; LOT 2154867; 1:500)
Mouse monoclonal anti-OPA1 (BD Biosciences; 612606; LOT 4164691; 1:1000)
Rabbit polyclonal anti-YME1L (ProteinTech; 11510-1-AP; RRID:AB_2217459; 1:1000)
Rabbit Polyclonal anti-STARD7 (ProteinTech; 15689-1-AP; RRID:AB_2197820; 1:1000)

Validation

CD140a-APC species reactivity: mouse; tested applications: flow cytometry; product citations: 56 https://www.thermofisher.com/antibody/product/CD140a-PDGFRA-Antibody-clone-APA5-Monoclonal/17-1401-81
Sca-1-FITC species reactivity: mouse; tested applications: flow cytometry, IF,IHC, WB; product citations: 90; https://www.thermofisher.com/antibody/product/Ly-6A-E-Sca-1-Antibody-clone-D7-Monoclonal/11-5981-82
Terr-119-PE species reactivity: mouse; tested applications: flow cytometry; product citations: 34; https://www.thermofisher.com/antibody/product/TER-119-Antibody-clone-TER-119-Monoclonal/12-5921-82
pan-acetylated-Lysine species reactivity: all (including mouse); tested applications: WB, IP, IHC, IF, ChIP, EISA; product citations: 416; https://en.cellsignal.de/products/primary-antibodies/acetylated-lysine-antibody/9441
Fatty Acid Synthase species reactivity: human, mouse; tested applications: WB; product citations: 29; https://www.cellsignal.com/products/primary-antibodies/fatty-acid-synthase-antibody/3189
CBP species reactivity: human, mouse, rat, monkey; tested applications: WB, IP, IF, ChIP, ChIP-seq; product citations: 28; https://www.cellsignal.com/products/primary-antibodies/cbp-d6c5-rabbit-mab/7389
AceCS1 species reactivity: human, mouse, rat, monkey; tested applications: WB, IP; product citations: 18; https://en.cellsignal.de/products/primary-antibodies/acecs1-d19c6-rabbit-mab/3658
Histone H3 species reactivity: human, mouse, rat, monkey; tested applications: WB, IHC, IF, FC, ChIP; product citations: 17; https://en.cellsignal.de/products/primary-antibodies/histone-h3-1b1b2-mouse-mab/14269
ACC1 species reactivity: human, rat, pig; tested applications: WB, IP, IHC, IF, ELISA; product citations: 31; https://www.ptglab.com/products/ACACA-Antibody-21923-1-AP.htm#datasheet
ACLY species reactivity: human, mouse, rat; tested applications: WB, IP, IHC, IF, FC, ELISA; product citations: 19; https://www.ptglab.com/products/ACLY-Antibody-15421-1-AP.htm#datasheet
TOMM20 species reactivity: mouse, rat, human; tested applications: WB, IP, IF, IHC(P), ELISA; product citations: 212; https://www.scbt.com/p/tom20-antibody-f-10 and species reactivity for TOMM20 antibody from Sigma-Aldrich: mouse, rat, human; tested applications: IHC, ELISA, IF, WB; https://www.sigmaaldrich.com/catalog/product/sigma/wh0009804m1?lang=de&region=DE
b-actin species reactivity: mouse, rat, human, avian, bovine, canine, rabbit; tested applications: WB, IHC (P), ELISA; product citations: 7415; https://www.scbt.com/p/beta-actin-antibody-c4
pan-acetylated Histone H3 species reactivity: tested in human and predicted in a wide range of organisms including mouse; tested applications: ChIP, ChIP-seq, DB, WB; product citations: 43; https://www.activemotif.com/catalog/details/39139/histone-h3ac-pan-acetyl-antibody-pab-1
pan-acetylated Histone H4 species reactivity: human and eukaryotes (including mouse); tested applications: WB, ICC< ChIP; https://www.merckmillipore.com/DE/de/product/Anti-acetyl-Histone-H4-Antibody,MM_NF-06-866?ReferrerURL=https%3A%2F%2Fwww.google.com%2F#overview
Histone H4 species reactivity: Cow, Human; predicted to work with: mouse and rat; tested applications: Flow Cytometry, IHC-P, IP, WB, ChIP, ICC/IF; product citations: 26; https://www.abcam.com/histone-h4-antibody-mabcam-31830-chip-grade-ab31830.html
Citrate Carrier species reactivity: human; predicted to work with: Mouse, Rat, Rabbit, Horse, Chicken, Guinea Pig, Cow, Cat, Gog, S. cerevisiae, D. melanogaster, Zebrafish; tested applications: WB; product citations: 1; https://www.abcam.com/slc25a1-antibody-ab99168.html
Histone H3K27me3 published applications: ChIP, ChIP-Seq, Native ChIP (PMID: 28973448), WB, IF, ELISA, RNA FISH; Reactivity: Human, Mouse, Wide Range Predicted; citations: 203: https://www.activemotif.com/catalog/details/39155/histone-h3-trimethyl-lys27-antibody-pab
Histone H3K27ac: published appications: ChIP, ChIP-Seq; Native ChIP (PMIDs: 24013198 & 28245924); WB; IF, IHC(FFPE); Proximity Ligation Assay (PLA); Reactivity: Budding Yeast, Human, Wide Range Predicted; citations: 23; https://www.activemotif.com/catalog/details/39133
LAMP2: species reactivity: Mouse; Recommended Applications: FACS, Immunofluorescence, Immunohistochemistry, Immunoprecipitation, Western Blot; citations: 22; https://dshb.biology.uiowa.edu/ABL-93
PDH1: species reactivity: Mouse, Rat, Cow, Human; tested applications: WB, ICC/IF, Flow Cytometry; citations: 59; https://www.abcam.com/pdha1-antibody-9h9af5-ab110330.html
CD44-PE: species reactivity: human, mouse; tested applications: Flow Cytometry (Flow), Immunocytochemistry (ICC/IF); citations: 173; https://www.thermofisher.com/antibody/product/CD44-Antibody-clone-IM7-Monoclonal/12-0441-82
CD29-PE: species reactivity: mouse, rat; tested applications: Flow Cytometry, ICC/IF; citations: 55; https://www.thermofisher.com/antibody/product/CD29-Integrin-beta-1-Antibody-clone-eBioHMb1-1-HMb1-1-Monoclonal/12-0291-82

CD45-PE: species reactivity: mouse; tested applications: Flow Cytometry; https://www.fishersci.se/shop/products/anti-cd45-ptprc-pe-clone-30-f11-invitrogen/15226014
CD34-FITC: species reactivity: mouse; tested applications: Flow Cytometry; citations; 119; https://www.thermofisher.com/antibody/product/CD34-Antibody-clone-RAM34-Monoclonal/11-0341-82
CD31-APC: species reactivity: mouse; tested applications: Flow Cytometry; citations: 80; https://www.thermofisher.com/antibody/product/CD31-PECAM-1-Antibody-clone-390-Monoclonal/17-0311-82
GCN5: species reactivity: human, mouse, tested applications: WB, ELISA, citations: 5; https://www.ptglab.com/products/KAT2A-Antibody-14983-1-AP.htm
FLAG: species reactivity: all; tested applications: Western Blotting , Immunohistochemistry, Immunofluorescence (Immunocytochemistry), Flow Cytometry, Chromatin IP; citations: 313; https://www.cellsignal.com/products/primary-antibodies/dykddddk-tag-d6w5b-rabbit-mab-binds-to-same-epitope-as-sigma-s-anti-flag-m2-antibody/14793
YME1L: species reactivity: human, mouse, rat; tested applications: WB, IP, IHC, IF,ELISA; citations: 41; https://www.ptglab.com/products/YME1L1-Antibody-11510-1-AP.htm
LONP: species reactivity: human, mouse, rat; tested applications: WB, citations: 1; https://www.sigmaaldrich.com/DE/de/product/sigma/hpa002192
PGAM5: species reactivity: human; tested aplications: WB, Immunocytochemistry (ICC/IF), citations: 2; https://www.sigmaaldrich.com/DE/de/product/sigma/hpa036978#
STARD7: species reactivity: human, mouse, rat; tested applications: WB, IHC,ELISA; citations: 5;https://www.ptglab.com/products/STARD7-Antibody-15689-1-AP.htm
OPA1: species reactivity: Human (QC Testing), Mouse, Rat, Dog, Chicken (Tested in Development); tested applications: Western blot (Routinely Tested), Immunofluorescence (Tested During Development); citations: 3; https://www.bdbiosciences.com/en-us/products/reagents/microscopy-imaging-reagents/immunofluorescence-reagents/purified-mouse-anti-opa1.612606
PARL: species reactivity: mouse, pig, rabbit, horse, dog, rat, human; tested applications: WB, mmunocytochemistry (ICC/IF); citations: 1; https://www.sigmaaldrich.com/DE/de/product/sigma/av44851

Eukaryotic cell lines

Policy information about cell lines

Cell line source(s)	HEK293T were used to generate lentivirus.
Authentication	HEK293T were purchased freshly from ATCC with appropriate authentication
Mycoplasma contamination	Cell lines are routinely checked for mycoplasma and cells used in this study tested negative.
Commonly misidentified lines (See ICLAC register)	HEK293T have been found to be contaminated with HeLa (Nelson-Rees et al., 1981) - however, cells were freshly purchased from ATCC.

Animals and other organisms

Policy information about studies involving animals; ARRIVE guidelines recommended for reporting animal research

Laboratory animals	C57BL/6N wild type male mice were used in this study.<br>Mice were 3-4 months old (young cohort) and 18-22 months old (old cohort). Information about the housing conditions of the mice is provided in the respective section of the manuscript.
Wild animals	The study did not involve wild animals.
Field-collected samples	The study did not involve field-collected samples.
Ethics oversight	Landesamt für Natur, Umwelt und Verbraucherschutz Nordrhein-Westfalen (LANUV NRW) approved the protocol used in this paper.

Note that full information on the approval of the study protocol must also be provided in the manuscript.

Human research participants

Policy information about studies involving human research participants

Population characteristics	all relevant information can be found in Corrigan et al., Cells Tissues Organs 207, 83-96 (2019)
Recruitment	all relevant information can be found in Corrigan et al., Cells Tissues Organs 207, 83-96 (2019)
Ethics oversight	all relevant information can be found in Corrigan et al., Cells Tissues Organs 207, 83-96 (2019)

Note that full information on the approval of the study protocol must also be provided in the manuscript.

ChIP-seq

Data deposition

☒ Confirm that both raw and final processed data have been deposited in a public database such as GEO.

☒ Confirm that you have deposited or provided access to graph files (e.g. BED files) for the called peaks.

Data access links<br>*May remain private before publication.*	NGS (RNA- and ATAC-seq as well as CUT&RUN) data that support the findings of this study are available at gene expression omnibus, GEO143580; link: https://www.ncbi.nlm.nih.gov/geo/query/acc.cgi?acc=GSE143580; token: ydyjusqkhjorder
Files in database submission	GSM4711140 H3K27AC Young Sample 1<br>GSM4711141 H3K27AC Young Sample 2<br>GSM4711142 H3K27AC Young Sample 3<br>GSM4711143 H3K27AC Old Sample 1<br>GSM4711144 H3K27AC Old Sample 2<br>GSM4711145 H3K27AC Old Sample 3<br>GSM4711146 H3K27ME3 Young Sample 1<br>GSM4711147 H3K27ME3 Young Sample 2<br>GSM4711148 H3K27ME3 Young Sample 3<br>GSM4711149 H3K27ME3 Old Sample 1<br>GSM4711150 H3K27ME3 Old Sample 2<br>GSM4711151 H3K27ME3 Old Sample 3<br>GSM5266506 H3AC Young Sample 1<br>GSM5266507 H3AC Young Sample 2<br>GSM5266508 H3AC Old Sample 1<br>GSM5266509 H3AC Old Sample 2<br>GSM5266510 H3AC Old Acetate Sample 1<br>GSM5266511 H3AC Old Acetate Sample 2
Genome browser session<br>(e.g. UCSC)	bigwig files to upload in IGV are available at: https://figshare.com/s/aeafa250723a74204f96

Methodology

Replicates	Three replicates per antibody per condition for H3K27ac and H3k27me3 and two replicates for H3ac per condition
Sequencing depth	seq depth was between 10-15 million reads per sample (CUT&RUN)
Antibodies	Histone H3 K27ac (Active Motif, 39133; LOT: 31416013)<br>Histone H3 K27me3 (Active Motif, 39155; LOT: 31814017)<br>Histone H3acetylation (Active Motif; 39139; LOT:28518008)
Peak calling parameters	Peaks were called using chromstaR in differential mode between Young and Old for each histone mark (H3K27ac, H3K27me3 and H3ac) with the binsize of 1000, stepsize of 500 and 15 as minimum mapping quality threshold
Data quality	we used strict mapping criteria and spike-in controls for normalisation as background is very low in CUT&RUN
Software	bowtie2, MarkDuplicates, chromstaR, edgeR

Flow Cytometry

Plots

Confirm that:

☒ The axis labels state the marker and fluorochrome used (e.g. CD4-FITC).

☒ The axis scales are clearly visible. Include numbers along axes only for bottom left plot of group (a 'group' is an analysis of identical markers).

☒ All plots are contour plots with outliers or pseudocolor plots.

☒ A numerical value for number of cells or percentage (with statistics) is provided.

Methodology

Sample preparation	For cell sorting, cells were harvested, resuspended in HBSS+ buffer and incubated for 45 minutes with the following antibodies from eBioscience: CD140a-APC (#17140181), Sca-1-FITC (#11598185), Terr-119-PE (#12592182), the Fixable Viability Dye eFluor 450 (#65086314; 1:10000) and the Life Technologies CD45-PE antibody (#A16325). Antibodies Cells were then washed twice with HBSS+ and were filtered through 35µm nylon mesh into 5ml sample tubes. The CD45-/TERR-119-/CD140a+/Sca-1+ population was sorted into an eppendorf tube containing 500µl a-MEM media, at 4°C. Compensation was done using UltraComp compensation beads (#01222242, Invitrogen,) and ArC beads (#A10346, Life technologies).<br>For FACS-based marker characterization of the young and aged MSCs, cells were stained as desribed above with the respective

antibodies and analyzed by flow cytometry, using the BD FACSCANTO II instrument (BD Biosciences). Data were collected using FACS-Diva software and analyzed using FlowJo software.
To measure mitochondrial mass, cells were stained with the MitoTracker Deep Red FM probe (Thermo Fisher # M22426) for 30 minutes at 37°C, under hypoxic conditions. They were then washed with PBS, resuspended in a-MEM medium without FBS and phenol red and stained with DAPI (Invitrogen) for dead-cell exclusion. Right afterwards, cells were analyzed by flow cytometry (BD FACSCANTO II, BD Biosciences). Data were collected using FACS-Diva software and analyzed using FlowJo software. -

Instrument	Cell sorting was performed using BD FACSAria IIu and BD FACSAria IIIu instruments (BD Biosciences), under low pressure sorting conditions (100μm nozzle and 20 psi sheath pressure). The BD FACSCANTO II instrument (BD Biosciences) was used for the characterization of the marker profile of MSCs and for the analysis of the MitoTracker stained cells.
Software	FlowJo
Cell population abundance	At least 10000 cells were collected for each analysis experiment.
Gating strategy	Both for cell sorting as well as for cell analysis FSC and SSC were used to gate out cell debris and cell clumps.

☒ Tick this box to confirm that a figure exemplifying the gating strategy is provided in the Supplementary Information.
