# Supplementary figures and images for: Chromatin remodeling due to degradation of citrate carrier impairs osteogenesis of aged mesenchymal stem cells
Source: Nat Aging. 2021 Sep 13;1(9):810–25. doi: 10.1038/s43587-021-00105-8 (PMC10154229; doi:10.1038/s43587-021-00105-8)

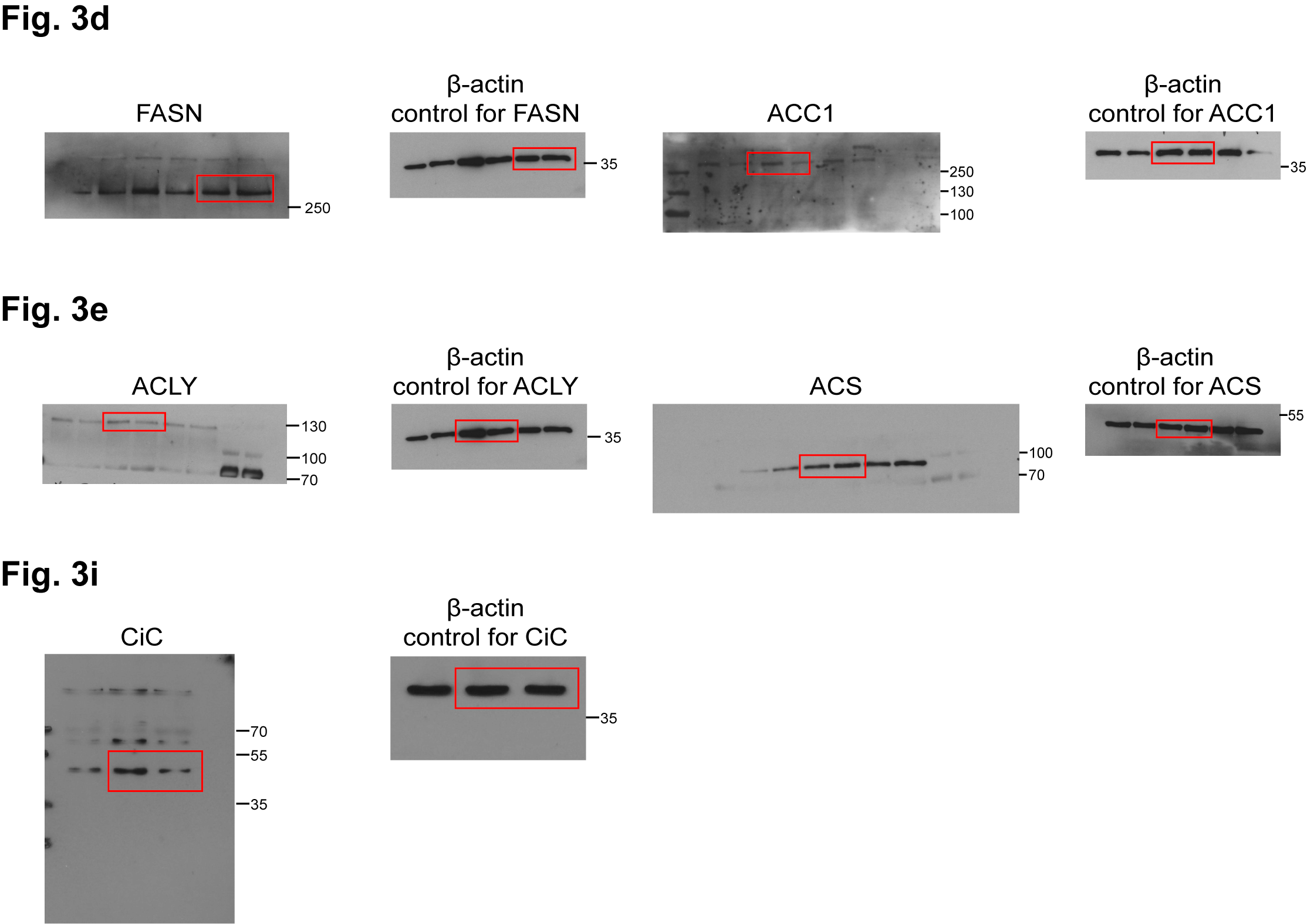

Supplement: Supplementary file 6 — Source Data Fig. 3. Unprocessed western blots for Fig. 3. [file 43587_2021_105_MOESM6_ESM.tif]

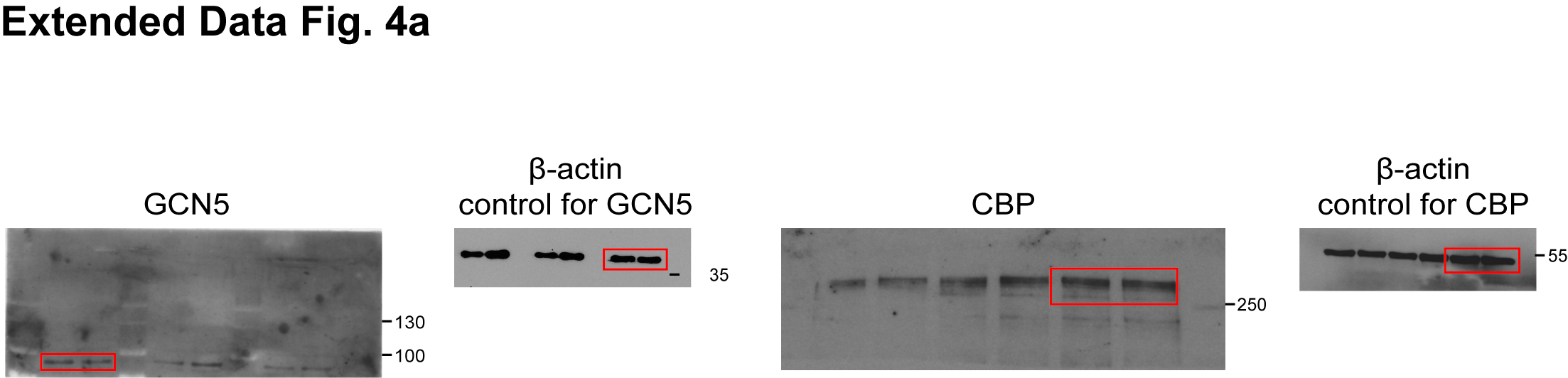

Supplement: Supplementary file 14 — Source Data Extended Data Fig. 4. Unprocessed western blots for Extended Fig. 4. [file 43587_2021_105_MOESM14_ESM.tif]

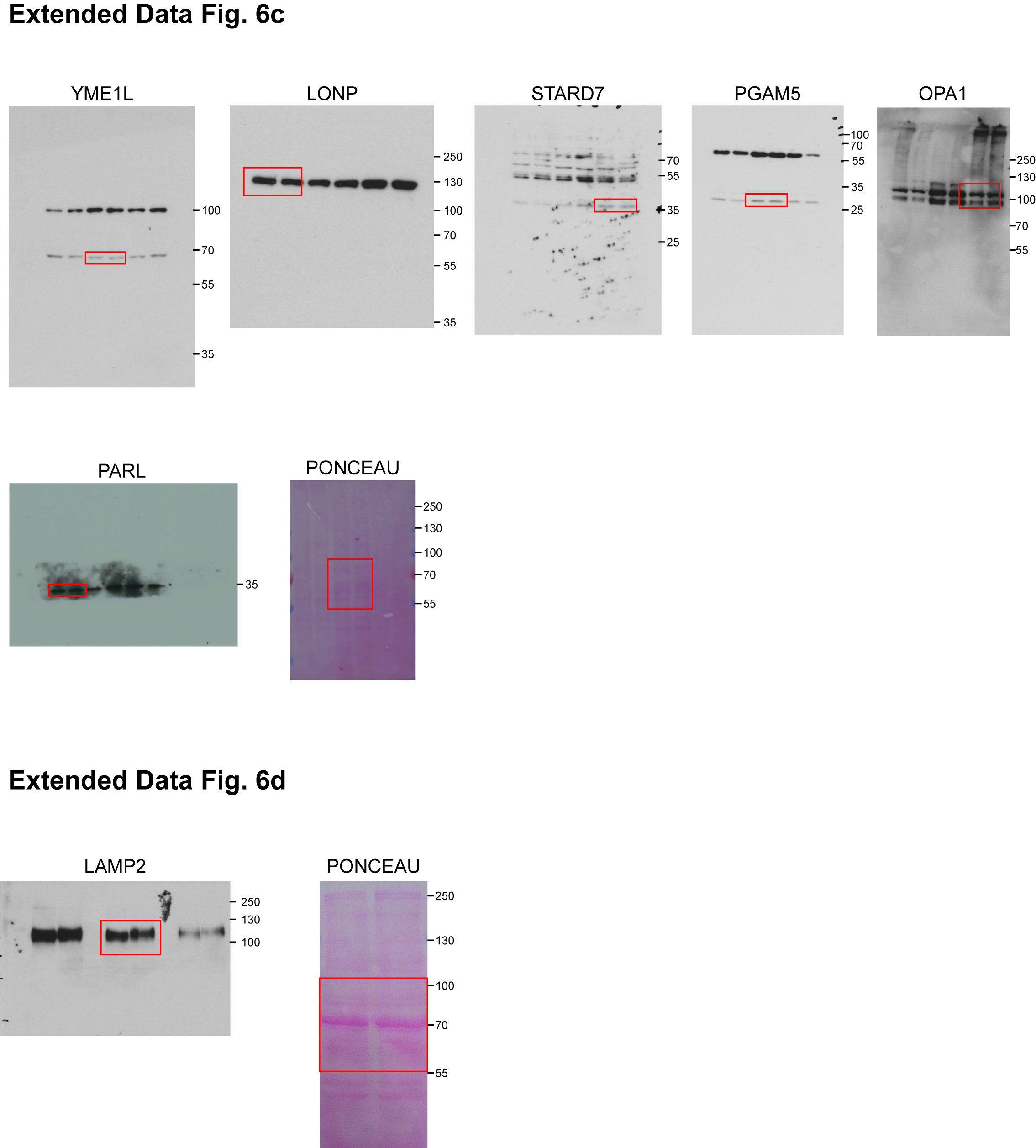

Supplement: Supplementary file 17 — Source Data Extended Data Fig. 6. Unprocessed western blots for Extended Fig. 6. [file 43587_2021_105_MOESM17_ESM.tif]
